# Deficits in Prediction Ability Trigger Asymmetries in Behavior and Internal Representation

**DOI:** 10.3389/fpsyt.2020.564415

**Published:** 2020-11-20

**Authors:** Anja Philippsen, Yukie Nagai

**Affiliations:** International Research Center for Neurointelligence (IRCN), The University of Tokyo, Tokyo, Japan

**Keywords:** predictive coding, computational modeling, recurrent neural networks, internal representation, autism spectrum disorder

## Abstract

Predictive coding is an emerging theoretical framework for explaining human perception and behavior. The proposed underlying mechanism is that signals encoding sensory information are integrated with signals representing the brain's prior prediction. Imbalance or aberrant precision of the two signals has been suggested as a potential cause for developmental disorders. Computational models may help to understand how such aberrant tendencies in prediction affect development and behavior. In this study, we used a computational approach to test the hypothesis that parametric modifications of prediction ability generate a spectrum of network representations that might reflect the spectrum from typical development to potential disorders. Specifically, we trained recurrent neural networks to draw simple figure trajectories, and found that altering reliance on sensory and prior signals during learning affected the networks' performance and the emergent internal representation. Specifically, both overly strong or weak reliance on predictions impaired network representations, but drawing performance did not always reflect this impairment. Thus, aberrant predictive coding causes asymmetries in behavioral output and internal representations. We discuss the findings in the context of autism spectrum disorder, where we hypothesize that too weak or too strong a reliance on predictions may be the cause of the large diversity of symptoms associated with this disorder.

## 1. Introduction

Predictive coding is a general theory that has been proposed as an underlying principle of cognitive processes in the brain (1–3). According to this theory, new bottom-up sensory signals are integrated with top-down prior predictions to form posterior perceptions. Such priors could be provided by internal models (4) which the brain creates in order to predict how sensory percepts are caused by the external world and our own actions. In the framework of predictive coding, perception and action selection can be interpreted as permanent efforts to minimize the prediction error (or to maximize the amount of information gained). Many recent studies demonstrated that computational models based on predictive coding can account for the development of sensorimotor control (5, 6), multisensory cue integration (7), and other aspects of human behavior and cognition (6, 8).

The relative reliance on predictions at the expense of sensory signals is an important parameter: attending to one's own predictions is necessary to exploit previous experiences, but attending to sensory signals is required for learning new and previously unobserved patterns. How humans integrate these two signals depends on the situational context and previous experiences. However, in addition, there might also be individual tendencies to rely more strongly on either predictions or sensory signals. These ideas have been recently investigated in the context of developmental or psychiatric disorders (9–14). In particular, many studies focus on autism spectrum disorder (ASD), a neurodevelopmental condition whose neurological origin remains unclear. Individuals with ASD exhibit deficits in social interaction and atypicalities in sensory processing. A particular challenge in studying ASD is its heterogeneous phenotype: Some people diagnosed with ASD show only slight aberrations from typical behavior, while others exhibit severe impairments of social interaction and language learning (15). It has been suggested that predictive coding could account for symptoms, such as hypersensitivities and the detail-focused processing style of individuals with ASD: Stronger reliance on sensory signals at the expense of prior predictions could alter information processing and affect perception and behavior (9, 10, 16, 17).

To investigate the plausibility of these claims, computational replications of common symptoms or other characteristics of the disorder are required. In particular, generative computational models have to be built that can test the effect of modifying specific aspects of the neural mechanisms on the model's behavior. In contrast to other computational approaches to autism that mainly aim at diagnosing ASD from behavioral data (18, 19), generative approaches like our study aim at identifying potential causes for impairments by manipulating model parameters that could be relevant for certain neural functions. Various models have been proposed recently for computationally replicating ASD and other disorders, such as schizophrenia using parameter modifications of generative models, in particular, using neural networks (11). A growing number of proposed models in recent years are based on predictive coding ideas as an underlying mechanism (20–25). One study that tested the effect of aberrant sensory precision was performed by Idei et al. (22). In their study, they investigated how aberrant sensory precision in a neural network affected the behavior of a controlled robot in interaction with a human experimenter. They found that repetitive behavior occurred with increased as well as with decreased sensory precision. Despite a similar effect on behavior, the underlying mechanisms in the network dynamics differed in both extreme conditions. These results suggest that evaluating computational models could give important insights about the potential mechanisms of such developmental disorders. However, a shortcoming of their study (22) is that the parameter of the pre-trained “mature” system was modified after training. Thus, the effect of differences in information processing during the development could not be measured. To uncover differences in the way that the computational model represents a task, it is crucial that the prediction deficit is already present at an early stage of learning.

In this paper, we address this issue using a computational model that is similar to the model from Idei et al. (22), with a number of substantial differences. First, in contrast to (22) we apply the parameter modifications during the learning process such that we can investigate how the modifications affect the development of internal network representations. Second, we investigate the effect of two different parameter modifications that are related to the hypothesis of aberrant prediction ability in ASD (9, 16). The first parameter expresses how strongly the network relies on sensory input as opposed to its own predictions. In particular, a too strong reliance on predictions at the expense of reliance on sensory output has been suggested to be related to symptoms, such as hypersensitivity in ASD (9). The second parameter corresponds to the aberrant sensory precision parameter proposed by Idei et al. (22) and models overestimation or underestimation of environmental noise, based on theories that aberrant precision may cause inflexible behavior in subjects with ASD (16, 26). Finally, in this paper, we investigate the effect on behavior and internal representation at a more abstract level. Specifically, we train recurrent neural networks for the task of predicting and reproducing simple two-dimensional trajectories. This procedure enables us to gain a general understanding of the properties of the acquired internal network representations that are difficult to analyze in more complex task settings. Specifically, we compare the network's ability to reproduce the learned patterns with the quality of the formed internal network representations. This 2-fold evaluation enables us to assess how the network's behavior relates to the learned internal representations—an important measure that might predict the network's general performance, including its performance on novel tasks.

In contrast to previous computational models, the aim of this paper is not the replication of experimental data. Instead, we aim at evaluating how parameter modifications that are discussed as causes for ASD affect the development in general, with the aim to generate new hypotheses and propose a new direction for future research in cognitive neuroscience.

In the remainder of this paper, we first introduce the computational model and the task that we use to study the problem (section 2). Then, we present our results (section 3). Finally in section 4, we discuss potential implications of our findings for developmental disorders. Specifically, we suggest that some aspects of ASD might be explained by a too weak or too strong reliance on predictions.

## 2. Computational Model of Predictive Coding

An essential cognitive ability underlying many complex tasks is the ability to learn patterns from observations of the environment and use them for predicting the future (6). Our computational model is based on a recurrent neural network (RNN) and learns to predict temporal dynamics and their statistics. During a training phase based on prediction error minimization (2, 6), the network learns to predict the sensory perception of the next time step. Therefore, the network takes the role of an internal model which represents the dynamics of the observed world (4).

The crucial ingredient of our model is an integration that happens at the input level of incoming sensory information and the predictions that the network produces in every time step. A network, thus, can make new predictions either directly from sensory input, or by utilizing its own output from the previous time step as a predicted input signal (or *prior*).

**Figure 1:**
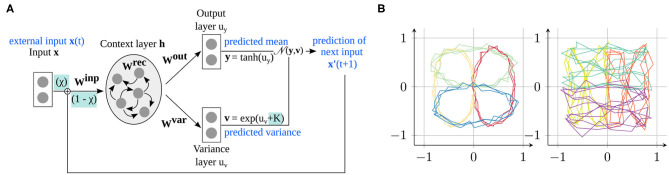
Model and data used in the experiments. **(A)** The S-CTRNN learns to predict input sequences by estimating the mean and variance of the next time step via a recurrent context layer. Our parameters modify how the network integrates external sensory information with its own predictions (χ*train*) and determine whether the variance is accurately estimated or overestimated/underestimated (*K*). **(B)** Training data provided to the network consisting of four ellipses (left) and four “eight” shapes (right), located at four different positions in the input space.

In this section, after introducing the model and the training procedure in section 2.1, we explain how the parameter modifications can be used to change the network's prediction ability (section 2.2). Then, the learning task is presented (section 2.3). An overview of the model is shown in Figure 1A.

### 2.1. Model

RNNs can learn to reproduce complex time-varying trajectories and are commonly used in neurocomputational studies and cognitive robotics experiments (27, 28). The particular network that we use is the stochastic continuous-time recurrent neural network (S-CTRNN) (29), a recurrent neural network model that is simple yet sufficiently powerful to implement the predictive coding paradigm. It has been previously applied for modeling ASD (22). The S-CTRNN differs from traditional RNNs in that it can predict the mean and the variance of time-fluctuating trajectory data $x_{0},x_{1},\ldots ,x_{t},\ldots ,x_{T}$ by capturing time-dependent information in the recurrent layer of the network $u_{t}$. The estimation of variance (inverse precision) is important, because it can be used to selectively update the prediction error arising between the sensory input and the network's own predictions. For example, a large prediction error is taken less into account when a large variance in the sensory signal is expected. This mechanism prevents the network from overlearning unpredictable data and also enables us to investigate the effect of modifying the network's variance estimation mechanism (cf. section 2.2). We chose the S-CTRNN because it allows us to investigate alterations of variance estimation and because it was frequently chosen in similar studies (11). Whereas other recent deep learning approaches, in particular generative models, may be able to similarly reproduce our findings, the simple nature of the S-CTRNN enables us to thoroughly investigate the internal representations formed during learning.

As shown in Figure 1A, the network consists of an input layer (two-dimensional), where the input x is provided in every time step, a context layer with *N* context neurons connected to each other to learn the time dependencies in the trajectories (here, *N* = 70), and two output layers for estimating the mean y and variance v of the input in the next time step.

The two-dimensional coordinates of the trajectory $x_{t}$ are fed as input to the network in every time step. The state of the recurrent $u_{t}^{\text{rec}}$ layer is updated, given the current input *x*_*t,i*_ (input dimension *i* in time step *t*), as

(1) $$\begin{array}{r} u_{t,i}^{\text{rec}}=\left(1-\frac{1}{\tau }\right)u_{t-1,i}^{\text{rec}}+\frac{1}{\tau }\left(\overset{I}{\underset{j=1}{\sum }}w_{ij}^{\text{inp}}x_{t,j}+\overset{C}{\underset{j=1}{\sum }}w_{ij}^{\text{rec}}h_{t-1,j}\right), \end{array}$$

where τ is a time scale parameter that determines how quickly the recurrent layer adapts to a new input (analogously to previous studies (22, 29), we set τ = 2). Further, *I* and *C* denote the dimensions of the input and context layer, respectively, and *w*_*ij*_ denotes the incoming synaptic weight of neuron *i* from neuron *j*. Neuron activations $h_{t}$ of the context neurons are computed from the network's state as $h_{t,i}=\text{tanh}\left(u_{t,i}^{\text{rec}}\right)$.

The states of the neurons in the output and output variance layer are updated from the context layer as

(2) $$\begin{array}{r} u_{t,i}^{\text{out}}=\overset{C}{\underset{j=1}{\sum }}w_{ij}^{\text{out}}h_{t,j},\;u_{t,i}^{\text{var}}=\overset{C}{\underset{j=1}{\sum }}w_{ij}^{\text{var}}h_{t,j}. \end{array}$$

The predicted mean and variance of the next time step can be computed from these states using activation functions. Using the exponential function for computing the variance ensures positive output values:

(3) $$\begin{array}{r} y_{t,i}=\text{tanh}\left(u_{t,i}^{\text{out}}\right),\;v_{t,i}=\text{exp}\left(u_{t,i}^{\text{var}}\right). \end{array}$$

The network is trained to minimize the prediction error, given a set of training sequences (see Figure 1B and section 2.3 for details on the training data). All the available input is presented to the network, and the network's prediction is compared to the actual sensory input of the next time step. In particular, the likelihood that the estimated mean $y_{t}$ and estimated variance $v_{t}$ accurately predict the next time step $x_{t+1}$ should be maximized. Mathematically, this can be implemented as the minimization of the negative log likelihood:

(4) $$\begin{array}{r} -\text{ln}\left(L_{out}\right)=\overset{T}{\underset{t=1}{\sum }}\overset{O}{\underset{i=1}{\sum }}\left(\text{ln}\left(2\pi v_{t,i}\right)+\frac{{\left(x_{t+1,i}-y_{t,i}\right)}^{2}}{2v_{t,i}}\right), \end{array}$$

where *T* is the number of time steps and *O* denotes the output dimensionality.

During training, all network weights are updated (**W^inp^**, **W^rec^**, **W^out^**, **W^var^** in Figure 1A), as well as the initial activation vector of the recurrent network layer of the first time step *t* = 0, the so-called *initial states*. Different initial states for each category of trajectory input make it possible for the network to generate different dynamics depending on the type of training trajectory. The initial states, together with the full context activation trajectory, correspond to attractors of the RNN; each attractor corresponds to one asymptotic behavior of the network (which, after successful training, become the trained trajectories) (30). These initial states are initialized as zero and automatically determined during training. As better separation of the trajectories is achieved with a stronger differentiation between trajectories, a second likelihood term is included in the loss function such that initial states do not diverge forever, but maintain a predefined distance from each other (29):

(5) $$\begin{array}{r} -\text{ln}\left(L_{init}\right)=\overset{S}{\underset{s=1}{\sum }}\overset{C}{\underset{i=1}{\sum }}\left(\text{ln}\left(2\pi v_{dist}\right)+\frac{{\left(u_{0,i}^{\left(s\right)}-{\hat{u}}_{0,i}\right)}^{2}}{2v_{dist}}\right), \end{array}$$

where *S* is the number of different trajectory classes that the network is trained with. This formula maintains the distance of the actual initial states $u_{0}^{\left(s\right)}$ from the mean initial state ${\hat{u}}_{0}$ at a certain variance level, determined by a predefined variance term *v*_*dist*_ [here, *v*_*dist*_ = 10, informed by prior work (29)].

Learning proceeds in epochs, and at the end of each epoch, the prediction error is minimized by updating all network weights and the initial states via the Adam optimizer (31), which performs stochastic gradient descent optimization. The network is implemented using the deep learning framework CHAINER (32).

A number of learning parameters of the RNN have to be determined. Owing to the relatively simple training task, we observed that the exact choice of the parameters does not crucially affect the results. In the presented results, the recurrent layer of the network consists of 70 neurons. In each learning epoch, a single batch of training data is processed, with one batch containing all eight trajectories 50 times^1^, generated as clean trajectories with additive noise (cf. Figure 1B and section 2.3). Training stops when the learning converges, or after 30, 000 epochs. For this purpose, every 100 epochs, we assess how the mean and standard deviation of the likelihood (which define the amplitude of the weight updates) developed during the previous 500 learning epochs. If the improvement in the mean likelihood falls below a threshold (< 0.001), and the likelihood does not substantially fluctuate anymore during different epochs (standard deviation < 0.05), convergence is assumed and learning is stopped.

### 2.2. Parameter Modifications

The parameters that we manipulate in the RNN model modify two different aspects of the network's learning mechanism:

1. how the network integrates sensory input with its own predictions (*external contribution* parameter χ*_train_*),
2. and to what extent the network's estimated variance fits the actual training data variance (*aberrant precision* parameter *K*).

These two parameters are highlighted in Figure 1A with a turquoise background.

#### 2.2.1. External Contribution Parameter

The external contribution parameter χ*_train_* ∈ [0, 1] determines how strongly the network relies on sensory input at the expense of its own predictions. The motivation for using this parameter is to alter how much information the network receives from the input signal and how much it uses its own previous experience (the predictions). An imbalance of these two signals, in particular a stronger reliance on predictions, has been suggested as a potential cause for hypersensitivity in ASD (9). The parameter χ*_train_* in the network has a comparable effect: it regulates how strongly the network utilizes sensory input for making predictions. In other words, modifying χ*_train_* changes how the network “perceives” the world.

This parameter change is implemented by changing the update formula of the context layer in Equation (1) such that the network receives the *integrated input* instead of the raw trajectory input:

(6) $$\begin{array}{r} x_{t+1,i}^{\prime }=\chi_{train}\cdot x_{t+1,i}+\left(1-\chi_{train}\right)\cdot y_{t,i}+\nu ,\;\nu \sim N\left(0,v_{t,i}\right), \end{array}$$

where *x*_*t*+1, *i*_ corresponds to the raw trajectory input for the next time step and *y*_*t,i*_ and *v*_*t,i*_ are the network's prediction of the mean and variance, respectively, obtained from the previous time step. χ interpolates between those two sources of information. Further, Gaussian-distributed noise with the amplitude of the estimated variance is added to the predicted mean to imitate the estimated noise properties of the actual trajectory input. The integrated input trajectory is also used in the likelihood function. Instead of comparing the current network output to the original signal L(x(t),y(t),v(t)), the likelihood function compares the output to the integrated signal $L\left(x_{t}^{\prime },y\left(t\right),v\left(t\right)\right)$.

Thus, this parameter models hypersensitivity or hyposensitivity to the sensory input. A high value of χ*_train_* implies strong attention to the sensory signal. With a small value of χ*_train_*, the network focuses strongly on its own prediction while ignoring the sensory input.

Note that χ*_train_* = 0 would result in L(y(t),y(t),v(t)), i.e., in a zero prediction error. The network would ignore the external input completely, assuming that its predictions are always true, and no learning would take place. Therefore, we use a minimum value of χ*_train_* = 0.1 during learning. During testing, this parameter can be used to switch the network behavior between open-loop (χ = 1, reactive generation) and closed-loop (χ = 0, proactive generation) control.

#### 2.2.2. Aberrant Precision Parameter

The second parameter that we modify is the aberrant precision parameter *K* [first introduced by Idei et al. (22, 33)], which implements the idea that ASD subjects might face difficulties in accurately estimating the variance of the sensory signal (10, 16, 26). The following formula replaces the estimation of variance in Equation (3):

(7) $$\begin{array}{r} v_{t,i}=\text{exp}\left(u_{t,i}^{\text{var}}+K\right)+0.00001. \end{array}$$

Here, *K* = 0, *K* > 0, and *K* < 0 result in normal estimation, overestimation, and underestimation of the trajectory variance, respectively. This parameter was first introduced in Idei et al. (33) and is inspired by aberrant precision theories suggested for ASD (16, 26). The idea is that because of an over- or underestimation of the variability of the environment, prediction errors are not sufficiently considered (in the case of overestimation) or too strongly minimized (in the case of underestimation). Too strong or too weak prediction error minimization might cause inflexibility in behavior and could, therefore, entail difficulties in social interaction. This theory is supported by the experimental findings of the computational model in work by Idei et al. (22, 33).

By using this parameter modification during training, instead of after training, as proposed in Idei et al. (22, 33), we can manipulate how strongly the network relies on the estimated variance during prediction error minimization. Increasing the estimated variance with *K* > 0 causes the network to overestimate the sensory noise. The network, thus, will not minimize the prediction error so strongly, which reduces the effect of learning. A decrease in the estimated variance with *K* < 0 leads to the opposite effect. The network underestimates the noise in its predictions and updates the prediction error even in cases where the input is affected by irregularities or noise in the patterns—the network “overlearns”.

We presented an initial evaluation of these two parameters in a previous study (34), which we extend here by additionally investigating generalization capability and extending the discussion.

### 2.3. Learning Task

In this study, we aimed to investigate trained networks in terms of their performance as well as the quality of the emerged internal representation. Measuring how well the internal representations of an RNN are structured is not a trivial task. Certainly, quality cannot be assessed globally but only in the context of a given task. Thus, the internal representation quality can be considered as the network's ability to represent task-relevant properties in the recurrent context layer.

For this reason, we train the network with a structured task that allows us to relate the emerged internal representation to the properties of the training data. Specifically, we consider the task of drawing, i.e., learning to predict and generate simple shape trajectories on a two-dimensional plane, as shown in Figure 1B. These training data comprise eight different shapes, namely four ellipse shapes and four “eight” shapes. Each trajectory contains 75 time steps with three repetitions of the drawn shape (e.g., three circles), which encourages the creation of stronger attractors in the dynamical system. We added Gaussian noise to the trajectories to model environmental uncertainty and to make the task more complex. Specifically, Gaussian noise with a different standard deviation per trajectory type is added: $\sigma_{\text{ellipse1}}^{2}=\sigma_{\text{ellipse2}}^{2}=0.001,\sigma_{\text{ellipse3}}^{2}=\sigma_{\text{ellipse4}}^{2}=0.003,\sigma_{\text{eight1}}^{2}=\sigma_{\text{eight2}}^{2}=0.005\text{and}\sigma_{\text{eight3}}^{2}=\sigma_{\text{eight4}}^{2}=0.007$.

Previous studies have shown that the internal activation patterns of the RNN are structured during learning according to the given input data. With the given task, the network can structure the input data according to three different criteria: the shape of the trajectory (ellipse or “eight” shape), the position of the trajectory (right, left, top, bottom), and the level of signal noise. As the training data are coded, here, in terms of their spatial coordinates, it can be expected that structuring occurs according to the position of the trajectory.

The data used here is computer-generated and only loosely inspired by behavioral data (studies investigating drawing and writing ability in developmental disorders (35–37)). However, the simple structure of the task facilitates the analysis of the emerged neural network structure, and we expect that properties found in networks trained on the abstract data will also emerge when training on other, more complex tasks. Confirming this conjecture is an important challenge of future work.

## 3. Experimental Results

To investigate how the prediction ability of the network affects the network's development, we trained networks under different learning conditions by modifying the two parameters introduced above (section 2.2). We performed two separate experiments: first, we evaluated the behavioral performance and compared it with the corresponding neural activation patterns, and second, we investigated the generalization capability of the trained networks.

For each experiment and both parameters, 10 individual trials were performed. In each trial, all the training conditions were tested: [0.1, 0.2…1] for χ*_train_* and [−8, −4, −2, 0, 2, 4, 8] for *K*. Thus, we trained 10 × 10 networks for the parameter χ*_train_* and 7 × 10 networks for the parameter *K*. To compare the results under different conditions more effectively and to exclude the influence of random weight initialization on the outcomes, the same 10 sets of initial weights (small random values) was used once for the networks of all parameter conditions. The effects of both parameters were tested independently: While changing χ*_train_*, we kept *K* constant and equal to 0, assuming normal variance estimation. While changing *K*, we set χ*_train_* to 1 such that the aberrant precision of the variance affected only the prediction error computation and not the network's input in the next time step. All the results and evaluations were averaged across the 10 individual networks trained under each condition.

### 3.1. Evaluating Behavior and Internal Representation (Experiment 1)

The networks were trained under all the training conditions for the eight trajectories shown in Figure 1B, as described in section 2.1. To evaluate whether the networks learned the given task, we let each trained network generate the trained trajectories after training in a proactive manner, i.e., in the absence of the external input. For this purpose, we set the activations of the recurrent layer according to the learned initial state of the desired shape and performed closed-loop generation by setting χ = 0. The performance was determined by the overall prediction error during trajectory generation and provided us with an estimation of how well the network can solve the task at a *behavioral level* (section 3.1.1).

To investigate the network's internal representations, we recorded the activations of the recurrent layer during trajectory generation for each trained network and each training sequence. The recorded matrices of size 70 × 75 (70 context neurons over 75 time steps) contain information on how the context neurons of the networks represent the learned tasks. Using these data, we analyzed which tasks are represented in similar or different ways in the network (section 3.1.2).

#### 3.1.1. Task-Specific Performance

**Figure 2:**
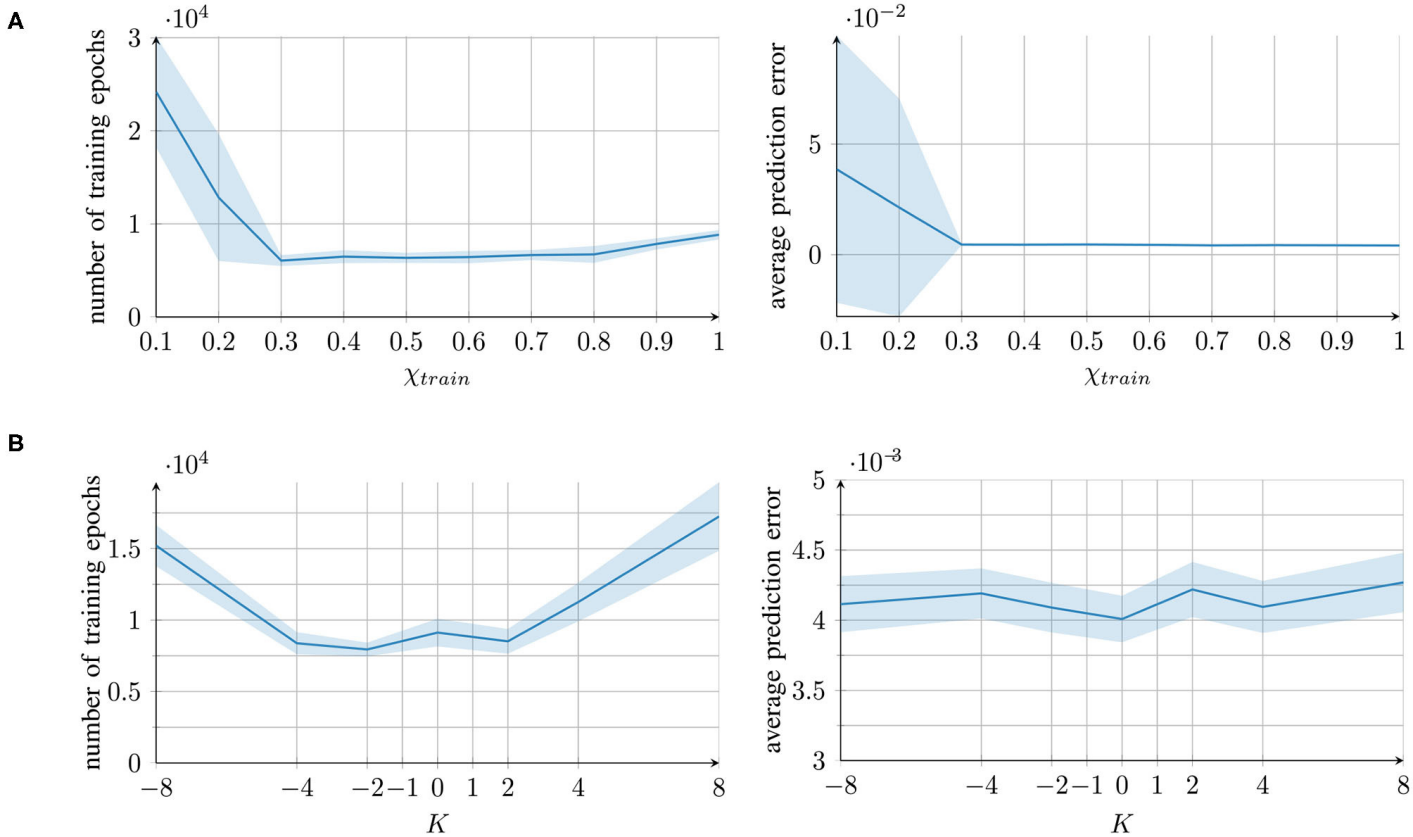
Training time and performance of the networks for different parameter conditions, measured as the number of training epochs (left) and the prediction error (mean square error) after training (right). For each parameter condition of **(A)** the external contribution parameter and **(B)** the aberrant precision parameter, mean and standard deviation of 10 individually trained networks are presented.

The networks' performances are shown in Figure 2 (right) along with the number of required training epochs (left). The prediction error refers to the mean-square error of the generated trajectories compared to one set of the training trajectories. Owing to the inherent noise of the training trajectories, a prediction error of 0.004 can be considered as optimal, as it corresponds to the average level of unpredictable trajectory noise.

For the parameter χ*_train_*, good performance was achieved under all the conditions except for extremely small values of χ*_train_* < 0.3. In these cases, the learning converged more slowly. The reason is that χ*_train_* affects the prediction error computation (see section 2.2). With a small χ, the integrated signal mainly comprises information from the network's own prediction and not from the original training signal, which causes a smaller prediction error. Therefore, smaller updates are performed and the learning converges more slowly.

With the parameter *K*, good performance was achieved under all the parameter conditions; however, a longer training time was required for extreme values of *K*. With overestimated variance (*K* > 0), the prediction error was reduced; thus, the network performed smaller weight updates. An underestimation (*K* < 0) caused excessively strong weight updates, which increased the training time owing to overshooting.

**Figure 3:**
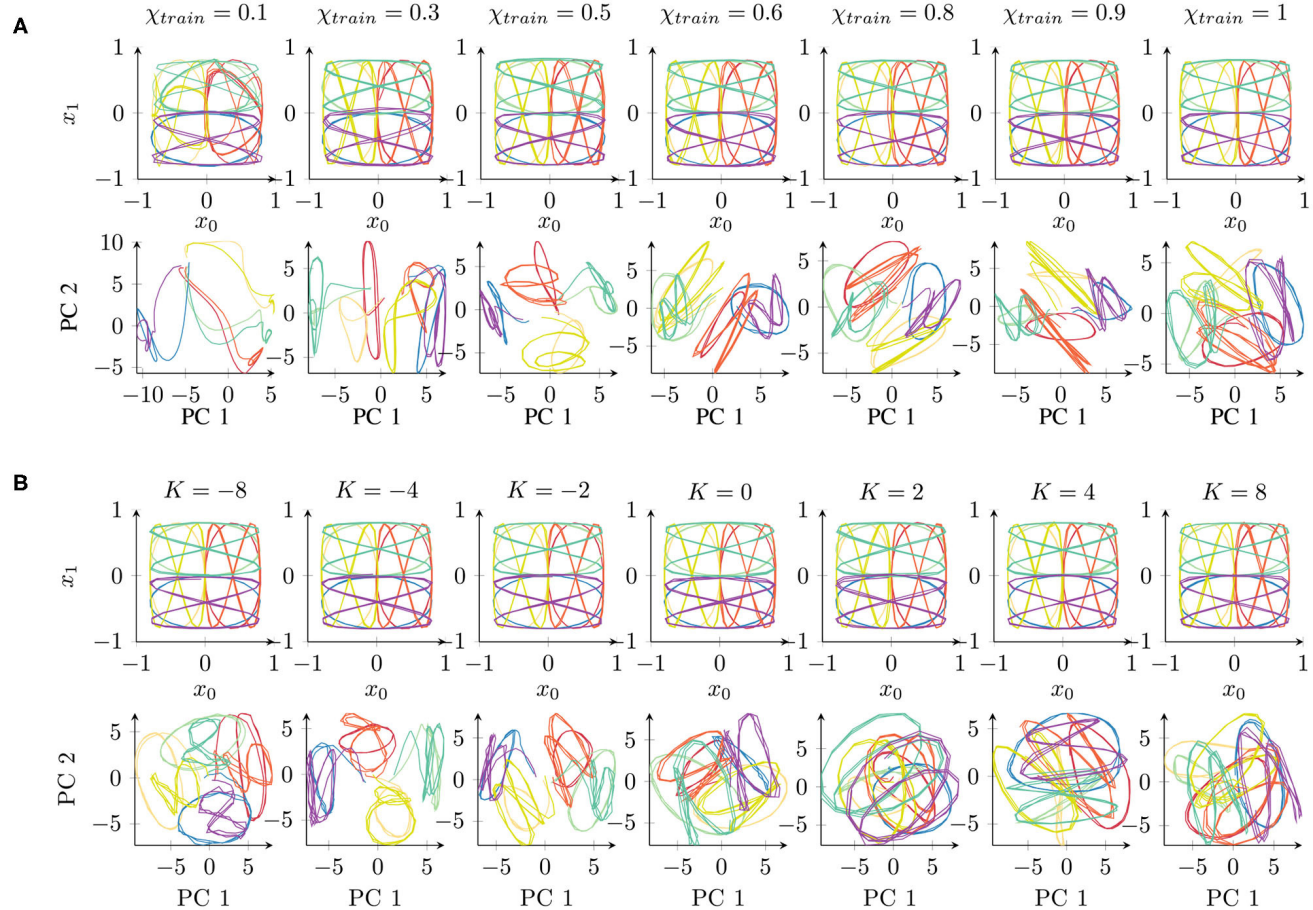
Qualitative comparison of networks trained under different parameter conditions of **(A)** χ*_train_* and **(B)** *K*, starting with the same initial weights. The top rows show the network's behavioral output, while the bottom rows show the first principal components (PCs) of the 70-dimensional space of the context activations. The colors correspond to the eight different trajectories (cf. Figure 1A). Readers may refer to the Supplementary Material for the results of all 10 trials.

In conclusion, all the parameter values, with the exception of extremely low χ*_train_* values, achieved successful learning. The networks were able to successfully recall the trained trajectories. The qualitative results of the reproduced trajectories can be seen in the top rows of Figures 3A,B.

#### 3.1.2. Internal Representation Quality

The recurrent context layer self-organizes during learning (via updates of the initial activations and the connection weights), and an analysis of the neural activations during pattern generation provides insights into how different tasks are structured in the network (38). Many RNN studies have investigated how task-specific features are reflected in the trained networks by measuring the activations of the context layer neurons while executing a task and they have suggested that such internal representation relates to the generalization capability (39, 40). This evaluation is similar to an assessment of activations of neuron populations via low-dimensional embeddings as it is commonly performed in neurological studies (41–43). It has been shown that such low-dimensional embeddings reflect specific features of the underlying task (43).

First, we performed a qualitative evaluation by projecting the per-time-step activations to two dimensions using principal component analysis (PCA). This visualization enables us to obtain an impression of how the network represents the given set of trajectories. Figure 3A shows an example of networks trained independently with different parameter values (starting from the same initial weight condition). Corresponding to the behavior (Figure 3A, top) shown in the two-dimensional task space, the internal representations are shown in the space formed by the first two principal components (Figure 3A, bottom) which capture about 43 or 41% of the overall variance in the χ*_train_* or *K* parameter condition, respectively.

The behavior outputs are similar under all the conditions, with only slight impairment for χ*_train_* = 0.1. By contrast, the corresponding internal representations change drastically. With χ*_train_* = 1, the internal representations for different trajectories overlap similarly to the original data shown in Figure 1B. With a smaller χ*_train_*, we find an increasing separation between the trajectories according to the position of the trajectories in space (similar colors denote the same position in the task space in Figure 3). Mathematically, such stronger separation is happening due to the feedback of the network's own prediction, which supports the generation of stronger attractors in the network. However, this only works with a sufficient contribution of the input signal. For this reason, extremely small values of χ*_train_* show a less well-structured representation. In particular, the activations do not show stable behavior but drift away while generating the three repetitions of a pattern.

The internal representations of an example of a set of networks for parameter *K* are shown in Figure 3B. There is no significant difference in the behavioral output (Figure 3B, top). In the internal representation (Figure 3B, bottom), we can observe a strong overlap of patterns if *K* > 0. By contrast, networks with *K* < 0 show a stronger separation of the patterns at the same position of the input space. A negative *K* causes an underestimation of the trajectory noise and, hence, stronger weight updates. Therefore, the weights are not accurately tuned and convergence takes longer. Positive *K* values overestimate the trajectory noise, which leads to careful weight updates. Interestingly, in terms of the training time, although overestimation and underestimation of the noise show a similar effect (Figure 2B), these two types of aberrant precision have different effects on the internal representation.

In summary, certain parameter conditions appear to cause a stronger separation of same-position trajectories in the space of context layer activation. As the input data code trajectories in terms of their positions, such structuring of the input space is beneficial and could, for instance, facilitate generalization capabilities, such as recognizing and generating new patterns (38).

To test whether the qualitative evaluations shown in Figure 3 also hold for the 70-dimensional context layer space, we performed a quantitative evaluation in the high-dimensional space of neuron activations. We computed two different measures by comparing the trajectories via the dynamic time warping distance (44). The distances between the activation time courses of patterns located at the same position in the input space (e.g., right circle and right “eight” shape) are averaged and called *inner distances*. The distances between the activation time courses of all the pairs of patterns located at different positions in the input space (e.g., left circle and right circle) are averaged and called *outer distances*. Good separation is achieved if the inner distances are small and the outer distances large; thus, a small quotient of these two measures (inner–outer quotient: inner distances divided by outer distances) indicates good separation.

**Figure 4:**
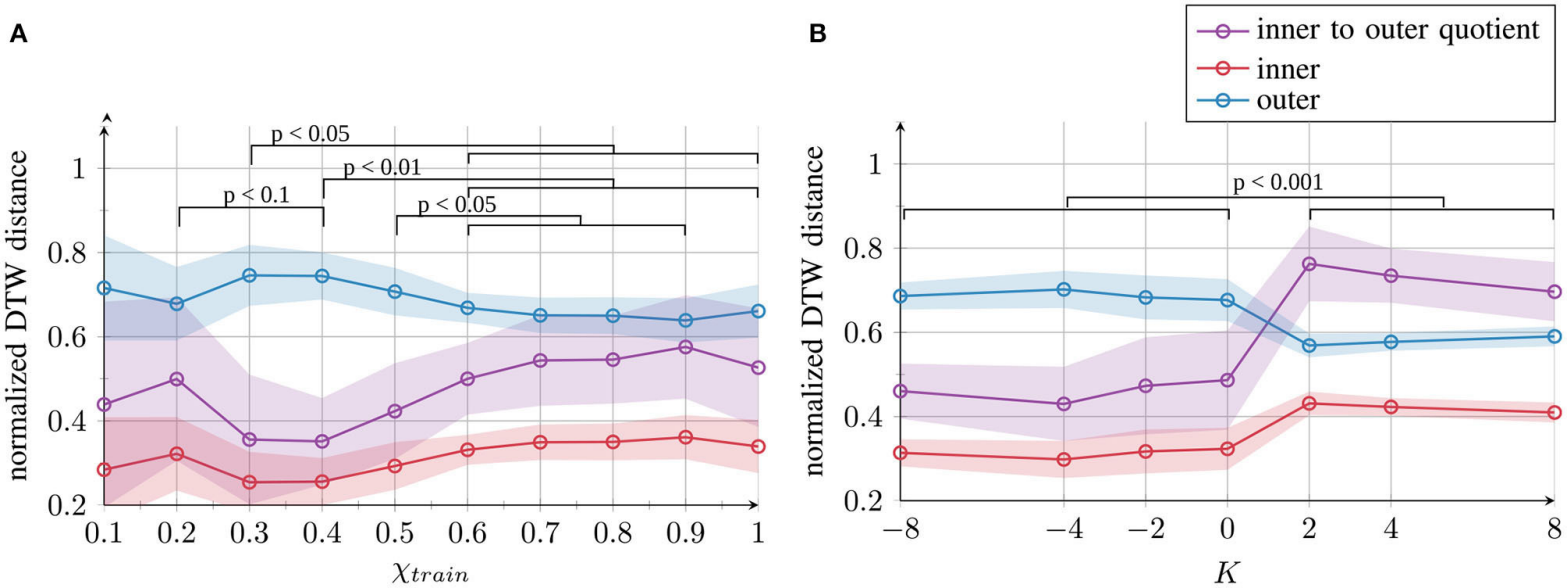
Quantitative results of the network's internal representation quality after training for **(A)** χ*_train_* and **(B)** *K*. Mean and standard deviation across 10 trials per parameter condition are presented. The inner and outer distances between the generated trajectories and the inner–outer quotient are measured as the dynamic time warping (DTW) distance. Statistical differences of the inner-outer quotient were evaluated on pairs of parameter conditions using the likelihood ratio test. Full tables of *p*-values are provided as Supplemental Material.

The results shown in Figure 4 confirm the qualitative observations: the best internal representation was obtained with χ*_train_* = 0.4. Furthermore, negative values, i.e., *K* ≤ 0, maintain good internal representation and positive values *K* > 0 lead to poor separation.

#### 3.1.3. Comparing Behavior and Representation Quality

**Figure 5:**
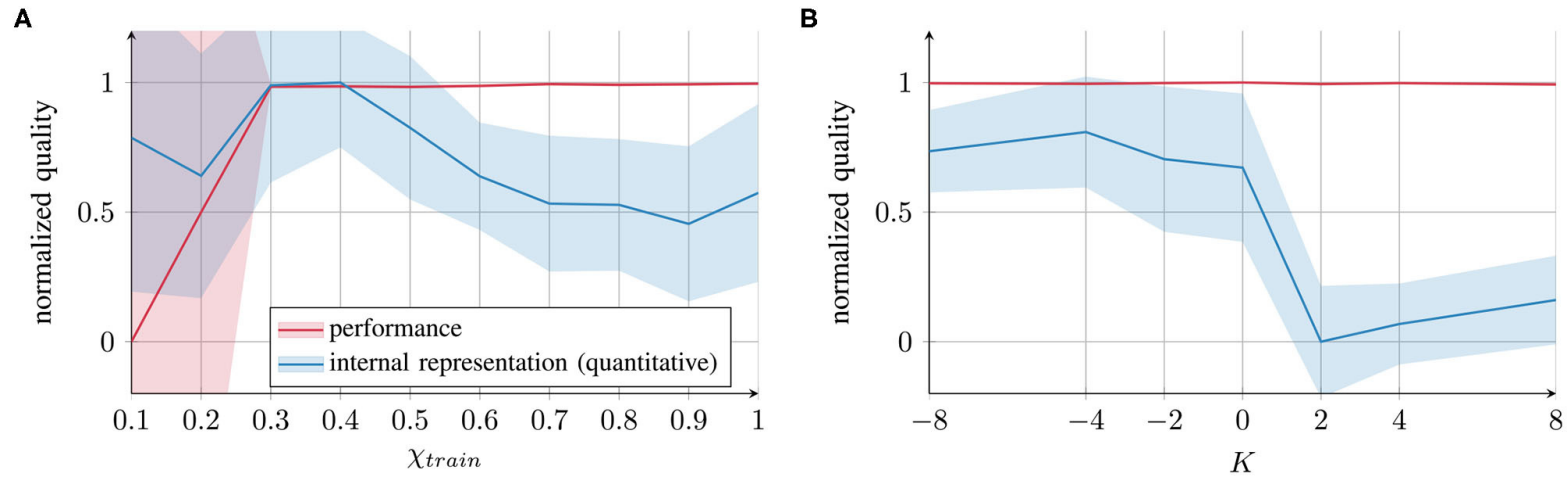
Comparison of task performance (evaluated as prediction error) and internal representation quality (evaluated via inner–outer quotient) for **(A)** χ*_train_* and **(B)** *K*. All the values are normalized across all the parameter conditions and subtracted from 1 (standard deviations scaled accordingly) to express a quality measure ∈ [0, 1].

The results of the behavioral performances and internal representations can be summarized as shown in Figure 5. The values were normalized across all the learning conditions for both parameters and flipped such that higher values correspond to better performance or better representation quality. It can be seen that all values of *K* and values of χ*_train_* ≥ 0.3 show good task performance with a small variance (red lines). The internal representation quality (blue lines) was optimal for moderate values of χ*_train_* and for *K* ≤ 0.

The first important conclusion that we can draw from these results is that for the external contribution parameter χ*_train_*, impairment of internal representation quality occurs at both ends of the parameter spectrum: the blue curve in Figure 5A exhibits an inverted U-shape, suggesting deficits at both extreme ends of the spectrum. However, the quality of impairment differs. With parameter values close to 0, the networks fail to form proper attractors and, therefore, coincide with behavioral deficits. With values close to 1, the networks form attractors (and the performance is intact); however, greater overlap occurs between the representations of different trajectories.

The second conclusion is that for χ*_train_* as well as for *K*, there is a gap between the behavioral performance and the internal representation quality. This result implies that the network's performance on trained data and the internal representation can be considerably different. Thus, behavior alone does not provide sufficient evidence for drawing conclusions about the network's underlying cognitive mechanisms. This finding indicates that neither behavior nor impairment in the internal network representation can be considered as a universal measure: assessing either of them does not predict the value of the other.

### 3.2. Generalization Capability to Recognize an Unknown Trajectory (Experiment 2)

The internal representation quality is often assumed to predict the network's generalization capability (38). To determine whether a good internal representation actually provides a practical benefit that becomes visible in the behavior, we performed an additional experiment. In this experiment, the networks were trained as before, but only on seven out of the eight trajectories shown in Figure 1B; the bottom “eight” shape (purple) was excluded. After training, we compared how well the networks perform in terms of generating the trained trajectories and generating the untrained trajectory that is new to the network.

For the untrained trajectory, none of the previously learned initial states can be used; therefore, a suitable initial state has to be determined from the high-dimensional space of the context activations. This inference process has been introduced as trajectory recognition in Murata et al. (45), and it proceeds by fixing the network weights after training and updating only the initial states (starting from a default position, e.g., the mean of all the initial states) while presenting an input trajectory to the network. Optimization is performed via backpropagation as described in section 2.1. After a sufficient number of epochs (here, 5, 000), the initial state converges to the value that best reproduces the given trajectory.

This initial state is then used as a starting point for generating the target trajectory via reactive generation (χ = 1). Thus, given the current time step, the networks perform only the prediction for the next time step.

**Figure 6:**
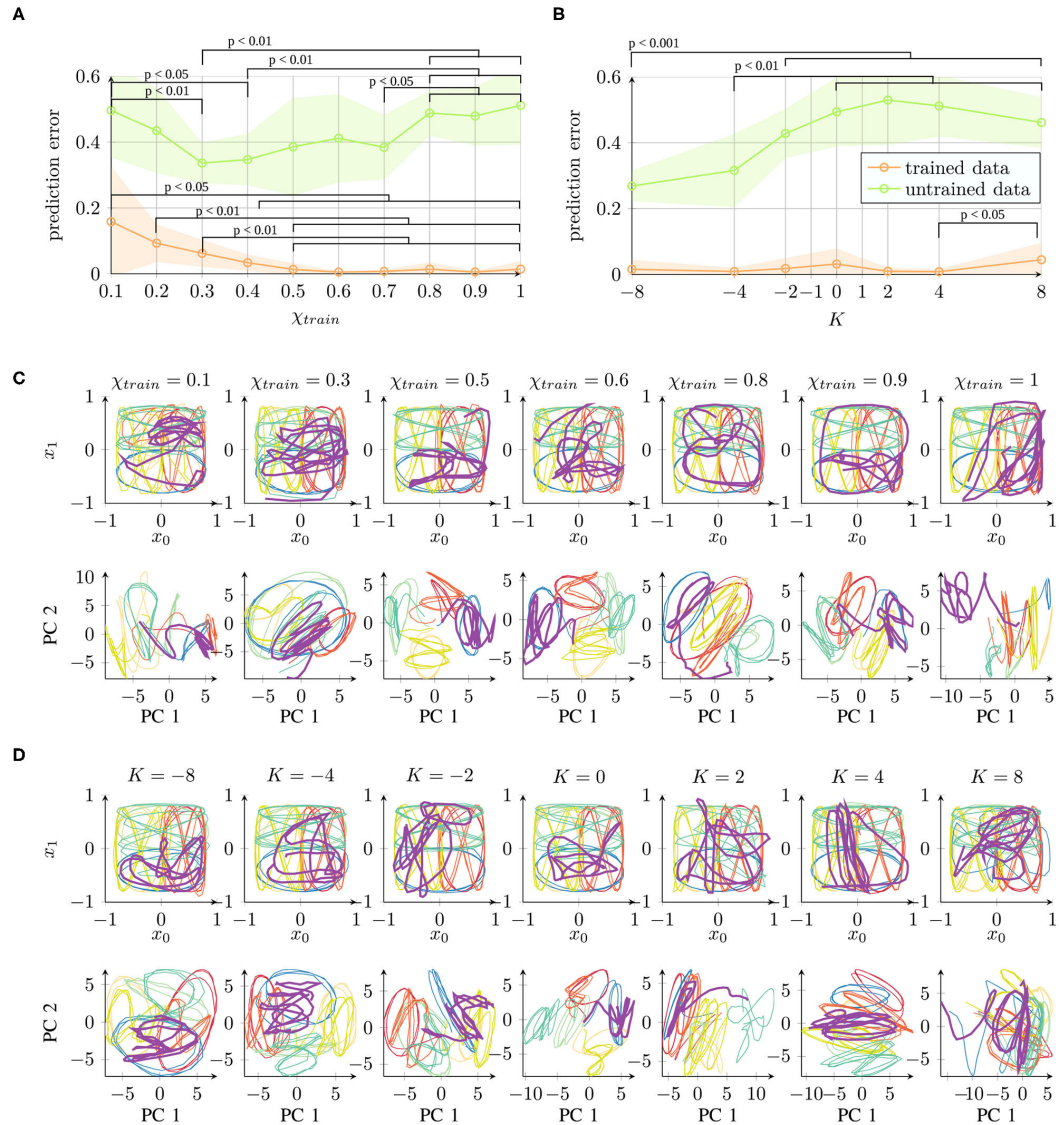
Network performance for trained data (training error) and untrained data (generalization capability) under different parameter conditions of **(A)** χ*_train_* and **(B)** *K* (prediction error (mean square error) and standard deviation averaged across 10 trials are shown). **(C,D)** Network performance output (top) and principal components of the context activations (bottom) during trajectory generation. The purple trajectory is untrained and thus corresponds to the generalization capability. Readers may refer to the Supplementary Material for the qualitative results of all 10 trials. Statistical differences in **(A,B)** on the trained/untrained data performance were evaluated on pairs of parameter conditions using the likelihood ratio test. Full tables of *p*-values are provided as Supplementary Material.

The results are shown in Figure 6. For parameter χ*_train_* (Figure 6A), a smaller prediction error on untrained data was achieved for values between 0.3 and 0.7 such that we can observe a U-shape. For parameter *K* (Figure 6B), better generalization was achieved for networks trained with a negative value of *K*. These findings correspond to the findings of internal representation quality.

In Figures 6C,D, qualitative examples are shown for the parameters χ*_train_* and *K*. The first and second principal components capture about 46 or 43% of the variance in the χ*_train_* or *K* parameter condition, respectively. It can be observed that the unknown purple trajectory overlaps with the blue (same-position) trajectory more commonly under conditions where better internal representation quality is measured.

**Figure 7:**
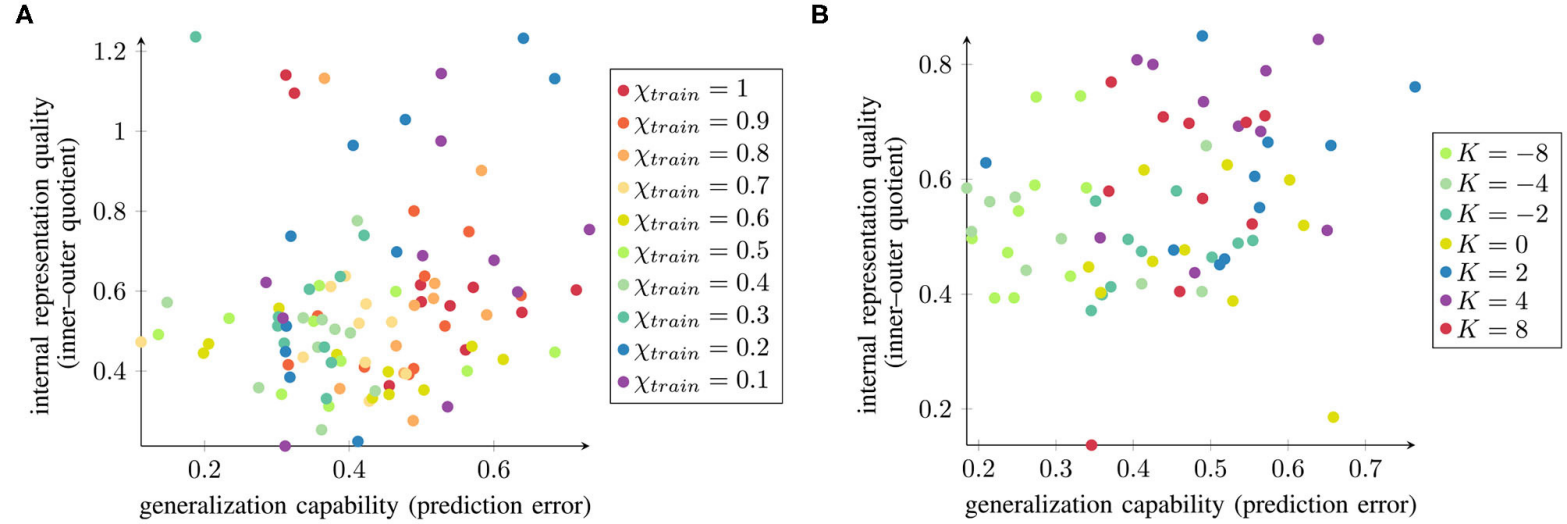
Normalized inner–outer quotient and the networks' generalization capabilities (small values are better in both cases) plotted against each other for all 10 trained networks under varying **(A)** χ*_train_* and **(B)** *K*.

To evaluate whether there is a network-wise correspondence between the internal representation quality and the generalization capability, we computed the correlations as shown in Figure 7. The parameter conditions with better internal representation quality are indicated in green. Networks trained with medium values of χ*_train_* and negative *K* are mainly located in the bottom left part of the spectrum, which represents better generalization and better separation in the context layer. However, the correlation between them (measured as the Pearson correlation coefficient) is only *r* = 0.13(*p* = 0.12) for χ*_train_* and *r* = 0.23(*p* = 0.06) for *K*, suggesting that there is too much variability among the data to find a clear correlation between these two measures. Nevertheless, there is a mild tendency indicating that networks with better internal representation quality are more likely to perform better for unknown trajectories and hence exhibit greater generalization capability.

## 4. Discussion

In this study, we evaluated how parametric modifications of the prediction ability of a computational model affect behavioral output and the quality of emergent internal network representations.

### 4.1. Summary of Experimental Results

The main findings of the experiments can be summarized as follows:

- Too strong as well as too weak attention to sensory input caused an impairment in the internal representation and generalization capability (U-shape). However, the quality of impairment is different and task-specific performances were opposite at both extreme ends of the parameter spectrum.
- Impairments at the neural level were not necessarily observable at the behavioral level, and vice versa. Thus, there appears to be no linear correlation between the behavioral performance and the internal representation quality.
- Despite high variability between different networks, there was a tendency for better generalization capability to be achieved with better internal network representation. Thus, internal representation quality appears to be an important measure that predicts performance in more general tasks.

These results indicate that modifying the model's prediction ability can have complex effects on the network's acquired capabilities. In particular, asymmetries are caused: a modification of moderate parameter values to either direction has different effects on internal representation and behavioral output. Differentiating behavior and internal representation, therefore, is important: impairments in internal representation may predict generalization capabilities, but does not always correspond to visible behavior in simple tasks like the execution of a movement.

### 4.2. Link to Computational and Psychological Studies of Developmental Disorders

In recent years, deficits in prediction ability have been frequently discussed as a potential underlying mechanism of developmental disorders, such as ASD (9, 16, 26). Therefore, our study might give some hints about how prediction ability shapes development and learning, and under which conditions disorders might emerge. In the following discussion, we review how the general mechanisms uncovered in this study relate to previous computational models of developmental disorders (section 4.2.1) and how they could translate to concrete experimental findings, using the example of ASD (section 4.2.2).

Our discussion on analogies with ASD is on purpose high-level oriented and rather highlights broad ideas than making specific connections to individual experimental findings. The reason is that this paper followed up on previous predictive coding literature, testing the effects of parametric modifications of prediction ability at a higher level using a generative approach. Specifically, we propose a generative model that allows us to study how neural impairments caused by the modifications of relevant parameters produce broad but systematic change in ASD behavior. We expect that these general findings of our study would replicate in more complex models and scenarios. However, future research is required to verify this expectation using more sophisticated network models to bridge the gap between computational modeling and experimental evidence from behavioral studies. A first step toward this aim has been performed in a recent study (25) which demonstrated that too weak and too strong reliance on priors can lead to two different types of impairment in a drawing completion task.

#### 4.2.1. Link to Existing Predictive Coding Models

In recent years, several computational studies have been performed to assess how impairments at a neural level might be related to impaired cognitive functions, especially, in the context of developmental disorders (11, 22–25). Various parameter modifications have been probed, many of which are related to prediction ability (22, 25). The findings in the present study extend previous findings, in particular of Idei et al. (22) which focused on the modification of the aberrant sensory precision parameter *K*. In Idei et al. (22), the authors modified the parameter after training and demonstrated that aberrant sensory precision in either direction may cause atypical behavioral characteristics. Whereas, the behavioral symptoms at both ends of the spectrum were comparable, different underlying mechanisms were found, namely the presence of either too strong or too weak prediction error signals. The authors suggest that an examination of the patient's neural activity may help to reveal how the atypical behavior is caused. The findings in our study support this suggestion: over- and underestimation of trajectory noise did not result in differences in task-specific performance but affected the internal representation in distinct ways. Thus, studying differences in the internal representations (i.e., the neural circuits in the biological brain) in addition to a behavioral evaluation could reveal additional insights that might not be visible at a behavioral level.

A crucial difference of our findings compared to Idei et al. (22) is that aberrant sensory precision did not cause an impairment at the behavioral level. The reason is that we applied the parameter modification not only after training but already during training, to account for the fact that aberrant information processing in developmental disorders should affect the model throughout the full course of learning. Our results indicate that, when aberrant sensory precision is already present during training, it does not necessarily cause an impairment of task-specific behavior: models with all values of *K* succeed to learn the trajectories and can reproduce them^2^. Thus, whereas Idei et al. (22) showed that a sudden increase or decrease of sensory precision causes atypical behavior, our findings suggest that when aberrant sensory precision persists throughout the lifetime, the model may be able to compensate the negative effects by experience. This finding may reflect the ability of many individuals with developmental disorders to acquire behavioral patterns that are indistinguishable from that of a healthy person. However, more time might be required for learning (cf. Figure 2) and impairments could arise when confronted with complex tasks which require them to generalize (cf. Figure 6).

#### 4.2.2. Analogy to ASD

In recent decades, ASD research shifted progressively toward a spectrum view on the disorder, moving away from the traditional study design that matched the performance of a group of ASD subjects with a group of typically developing (TD) individuals. It is well-known that autistic traits are also widespread in the general population (46, 47). Furthermore, many subjects with ASD have normal general intelligence and overcame limitations in their capabilities by practicing social communication. The performance of ASD subjects on specific tasks, thus, can exhibit large individual differences. While the concrete neural mechanisms remain unclear, various theories have developed over the decades to pinpoint the underlying causes of ASD (48, 49). Most recently, the so-called hypo-prior theory (9, 50) suggested that weak reliance on predictions may account for symptoms of ASD. In fact, a weaker reliance on prediction signals, or equally, a stronger relative reliance on sensory signals (14, 16) can replicate reduced susceptibility to visual illusions or hypersensitivity symptoms that have been found in many ASD individuals (47, 51–53). It may also explain intact imitation or overimitation (54) in ASD subjects, and superior performance due to stronger attention on sensory signals (55). However, some studies also find deficits in motor imitation (54), and numerous other symptoms (35, 36, 56) for which a hypo-prior theory cannot easily account.

**Figure 8:**
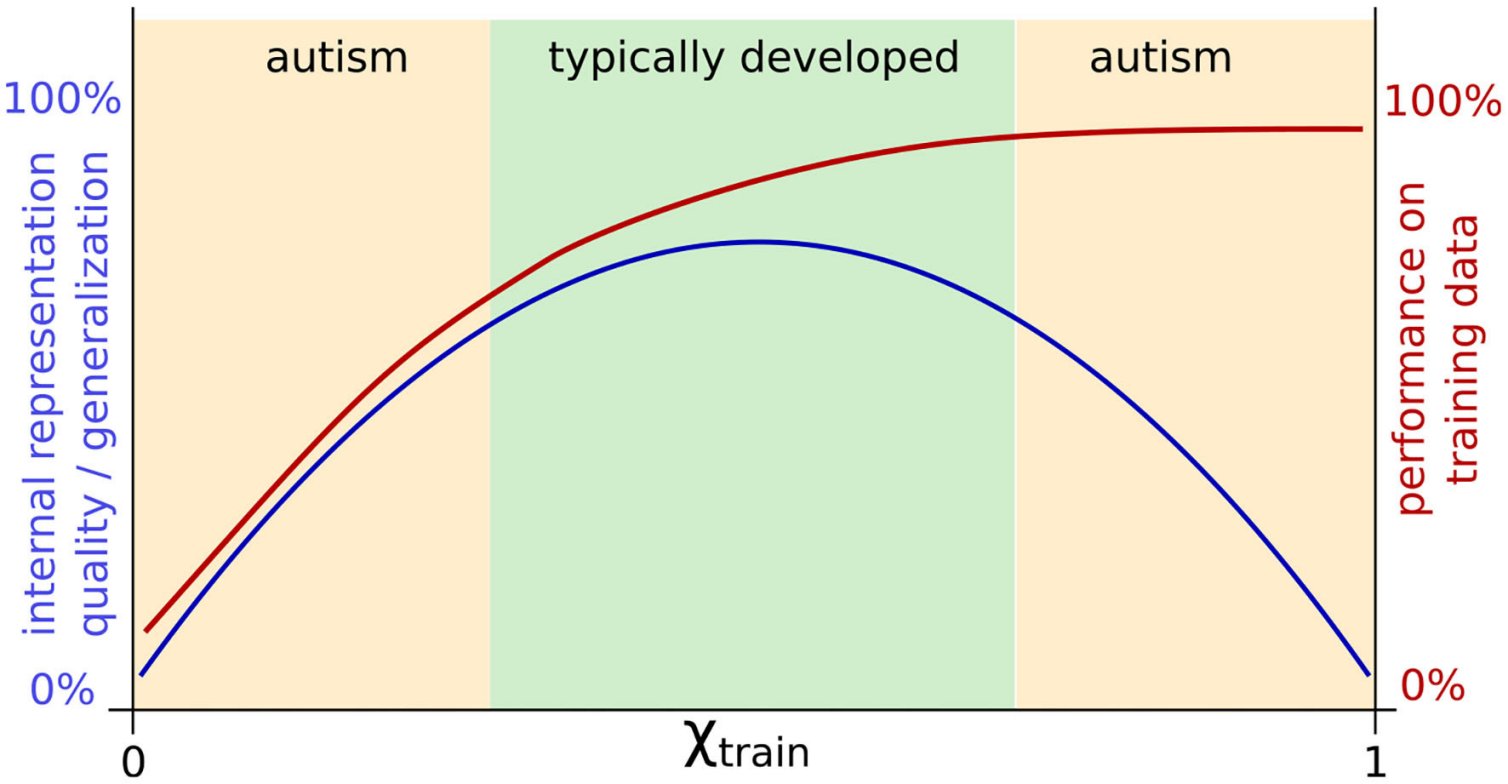
Schematic summary of the results for adjusting χ*_train_* and a possible interpretation: TD might correspond to medium parameter values with intact internal representation, whereas a failure to properly optimize performance and too strong optimization of task performance might impair internal representation quality. Both extremes of this spectrum could correspond to two different types of ASD.

The external contribution parameter χ*_train_* in this study was designed following the hypo-prior theory. With χ*_train_* close to 1, the network ignores its own predictions, focusing almost entirely on sensory signals for learning. As a result, we find intact performance, but an overlap in network representations constituting an impairment in internal representations that might implicate generalization deficits. As generalization deficits are commonly associated with ASD, the findings, thus, are in line with the predictions of the hypo-prior theory. However, we found impaired internal representations not only with a large χ*_train_*, but also with a small χ*_train_*. In contrast to a large χ*_train_* where task-specific performance was intact, with a small χ*_train_*, the impairment was additionally visible at the behavioral level. Therefore, not only weak reliance on predictions, but also atypically strong reliance on predictions, e.g., a “hyper-prior,” could lead to ASD-like characteristics. Following this interpretation, ASD could be characterized by an aberration from typical development in either direction. The important implication is that ASD might have two broad and different subtypes which might differ significantly and show even some seemingly incompatible behavioral symptoms. Moreover, this view implies that also TD is part of the same continuous spectrum in which differences in behavior and internal representation are modulated by continuous changes in the underlying mechanism. The idea is conceptually shown in Figure 8.

Although this is just a speculation at this point, it fits with observations in the clinical literature of ASD, in particular, with some inconsistent findings in behavioral studies of ASD. For example, despite realistic drawing abilities in many individuals with ASD (57–59), common deficits in drawing and handwriting have been reported (35–37, 56). Furthermore, studies on gaze behavior in TD and ASD have yielded conflicting results: some studies have suggested that individuals with ASD exhibit atypical gaze processing (60, 61), whereas many studies acknowledge that gaze behavior in ASD appears to be typical^3^, showing a stronger focus on the eye region (62, 63). In particular, despite the high variability exhibited by the ASD population in most studies, a tendency toward the extremes has been noted (64), for example, in low-level sensory processing (65–67), where opposite effects may occur between individuals or even within the same individual depending on the situation (68–70). In particular, studies have reported increased sensitivity, i.e., *hypersensitivity*, to certain sensory percepts, as well as seeming indifference to some sensory stimuli, i.e., *hyposensitivity* (51, 71). Our findings suggest that the same underlying mechanism could explain such aberrations in both directions.

In Figure 8, such opposite findings would be explained by the assumption that individuals with ASD differ from TD individuals in either direction, resulting in an impairment at the neural level and difficulties in generalization in a similar manner, but leading to opposite task-specific behavior. At one end of the spectrum (right side of Figures 4A, 6A, 8), we might find extraordinary skills, such as realistic drawing ability, which could result from overfitting for a particular task. The performance is extremely high, perhaps even superior to TD individuals, but the capability to solve general tasks may be impaired. Similarly, networks trained with χ*_train_* close to 1 exhibit overfitting, showing good specific performance but poor generalization. At the other end of the spectrum (left side of Figures 4A, 6A, 8), owing to underlearning, the networks cannot sufficiently attend to the sensory information (χ*_train_* close to 0), similar to how some children with ASD struggle to learn handwriting (35–37, 56) and sometimes show hyposensitivity to external stimuli (51, 71). Thus, opposite effects, such as hypersensitivity and hyposensitivity might originate from extreme impairments of the same underlying mechanism in either direction and should therefore not be considered in isolation but evaluated together. Hypersensitivity and hyposensitivity might be even causally related; for example, too much sensitivity to the environment could trigger an ASD individual to completely shut down his/her attention and to ignore the environment in order to reduce the stress level.

Furthermore, finding (ii) in section 4.1 states that behavioral performance does not necessarily correlate to impairments in internal representations. Therefore, an impairment at the neural level might not be observable directly in the behavior, and it only affects the generalization capability when confronted with more complex tasks. Thus, it cannot be inferred from typical performance that also the underlying cognitive mechanisms are typical; similar behaviors can be caused by different cognitive strategies. Such differences in cognitive mechanisms are to be expected not only between TD and ASD but also between different ASD individuals, and might in particular be used by individuals located at different ends of the spectrum as discussed above. For the example of gaze processing, more attention to the eye region can be caused by different cognitive strategies employed by subjects with and without ASD, which lead to similar observed behavior (72). For instance, eye contact can be motivated by a desire for social communication, but it can also reflect a drive to direct stronger attention to more salient features of the face (eyes have high contrast and pronounced movements, which attract attention). Hence, it is difficult to interpret the behavior of a subject with ASD without simultaneously measuring their internal representation.

In conclusion, aberrant prediction ability can cause complex effects on performance and internal representation quality and, therefore, is a good candidate mechanism that could aid to understand the heterogeneous literature of ASD research. A particular suggestion from our simulation results that could be tested in future cognitive neuroscience studies is that not only weak reliance but also overly strong reliance on predictions may be related to autistic traits.

There are, however, also a number of shortcomings of the current study. In particular, we assumed that one network, modeling one individual, is characterized by a fixed parameter value. However, individuals likely have the ability to adjust such a parameter value according to environmental and physiological changes (68, 69) There is some evidence that hyper- as well as hypo-sensitivity can even occur in a single individual (70). A recent study by Harris et al. (73) demonstrated that situational factors (specifically, the training protocol) influence learning in ASD. They showed that inflexible behavior in ASD caused by overlearning can be eliminated by reducing stimuli repetition, thus, by making the task more heterogeneous (i.e., less predictable). With this training protocol, ASD (as well as TD) subjects generalized better. One possible explanation that our study provides in this context is that the more heterogeneous task might have prompted the participants to adapt their internally used χ*_train_* parameter to a more moderate value to adapt to this situation. Further research should address how parameter values can be adapted flexibly according to the situation and task and which differences might exist between ASD and TD. To this end, ASD might be characterized by a tendency to use extreme values (64) or a reduced speed of updating (74, 75).

A further limitation is the relatively simple task setting in which we test the parameter settings. The reason for choosing such a task, here, was to investigate the general effects of the proposed parameters on development and to evaluate the internal representations in a systematic manner. Although our computational model simplifies cognitive development and considers behavior and representation quality only at an abstract level, we believe that this approach may open up new potential routes for discovering general features underlying the spectrum of ASD. We hope that these findings help to establish a basis for developing future models as well as for designing behavioral studies on ASD.

## Author Contributions

YN and AP conceived the experiments, analyzed, and interpreted the results. AP implemented and conducted the experiments, evaluated the results, and drafted the manuscript. YN reviewed the manuscript. Both authors read and approved the final manuscript.

## Conflict of Interest

The authors declare that the research was conducted in the absence of any commercial or financial relationships that could be construed as a potential conflict of interest.

## Footnotes

^1^Choosing a larger batch size mainly serves the purpose of improving the training efficiency on a GPU, as a single batch can be processed in parallel.

^2^In fact, generalization capability seems to be improved when the trajectory noise is underestimated and weights are strongly updated (cf. Figure 6). However, further research needs to clarify whether this tendency holds also for tasks with more complex noise characteristics.

^3^Note, that typical behavior in ASD might also arise due to explicit behavioral training (cf. applied behavior analysis).
