# Supplementary figures and images for: Deficits in Prediction Ability Trigger Asymmetries in Behavior and Internal Representation
Source: Front Psychiatry. 2020 Nov 20;11:564415. doi: 10.3389/fpsyt.2020.564415 (PMC7716881; doi:10.3389/fpsyt.2020.564415)

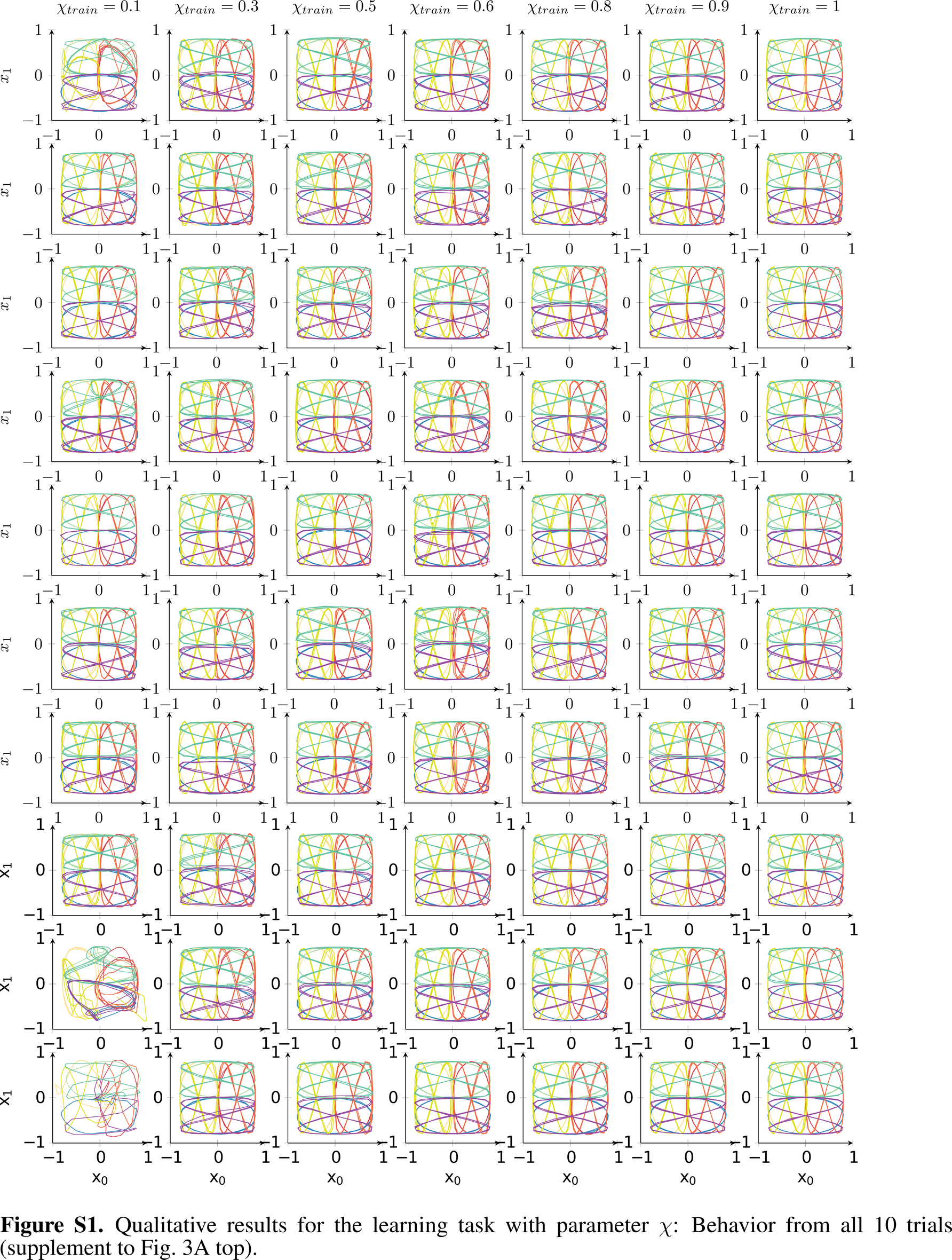

Supplement: Supplementary file 1 [file Image_1.TIF]

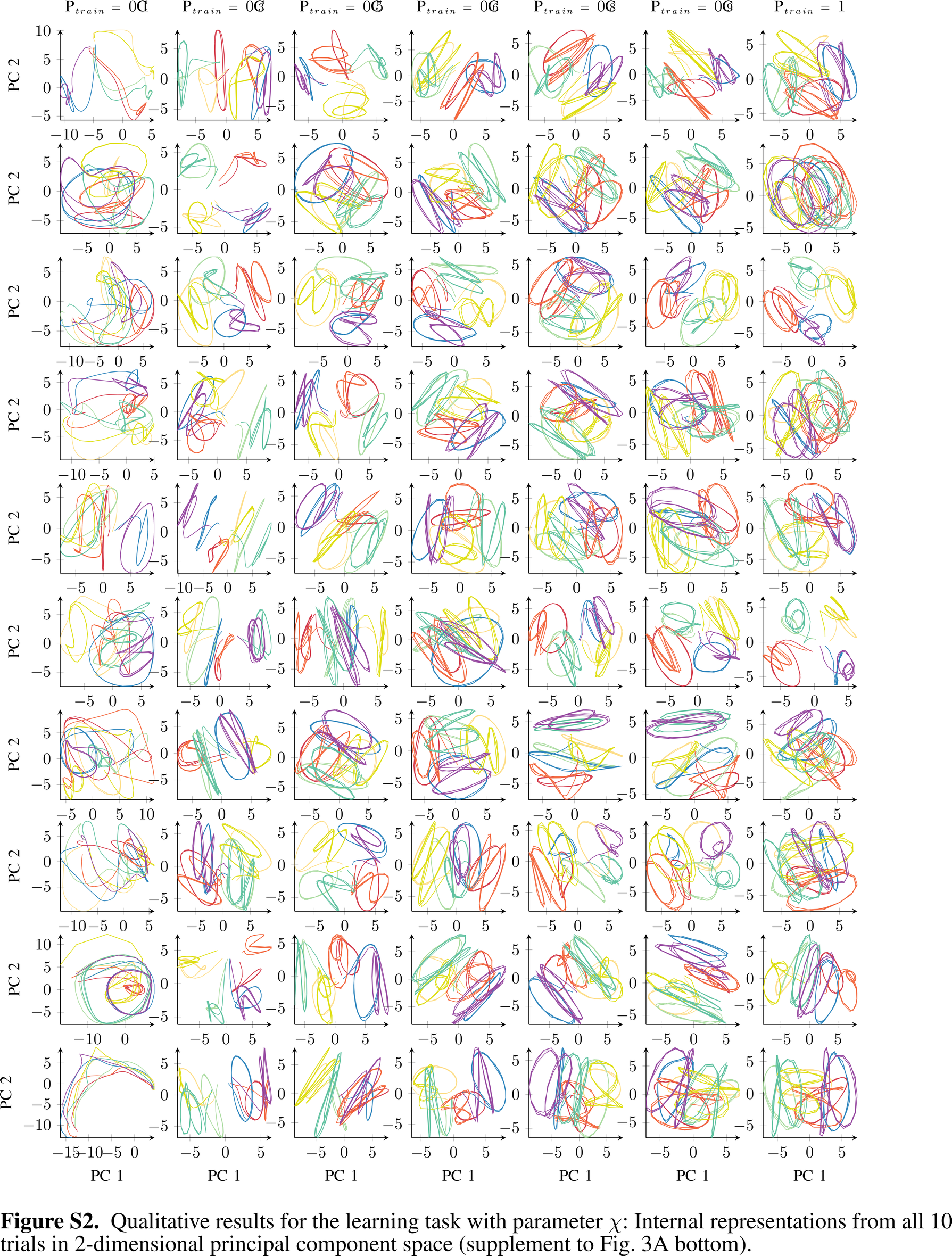

Supplement: Supplementary file 2 [file Image_2.TIF]

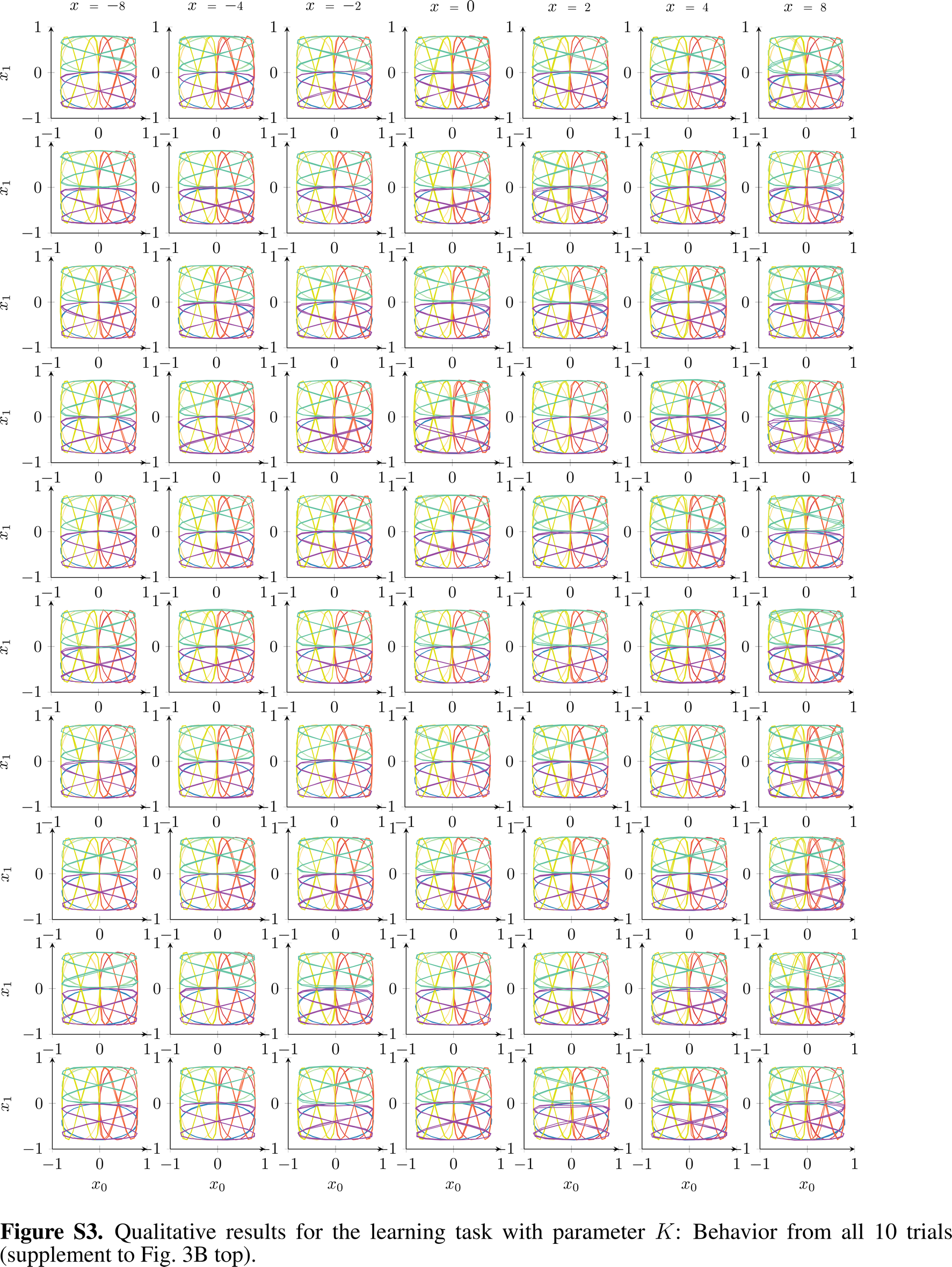

Supplement: Supplementary file 3 [file Image_3.TIF]

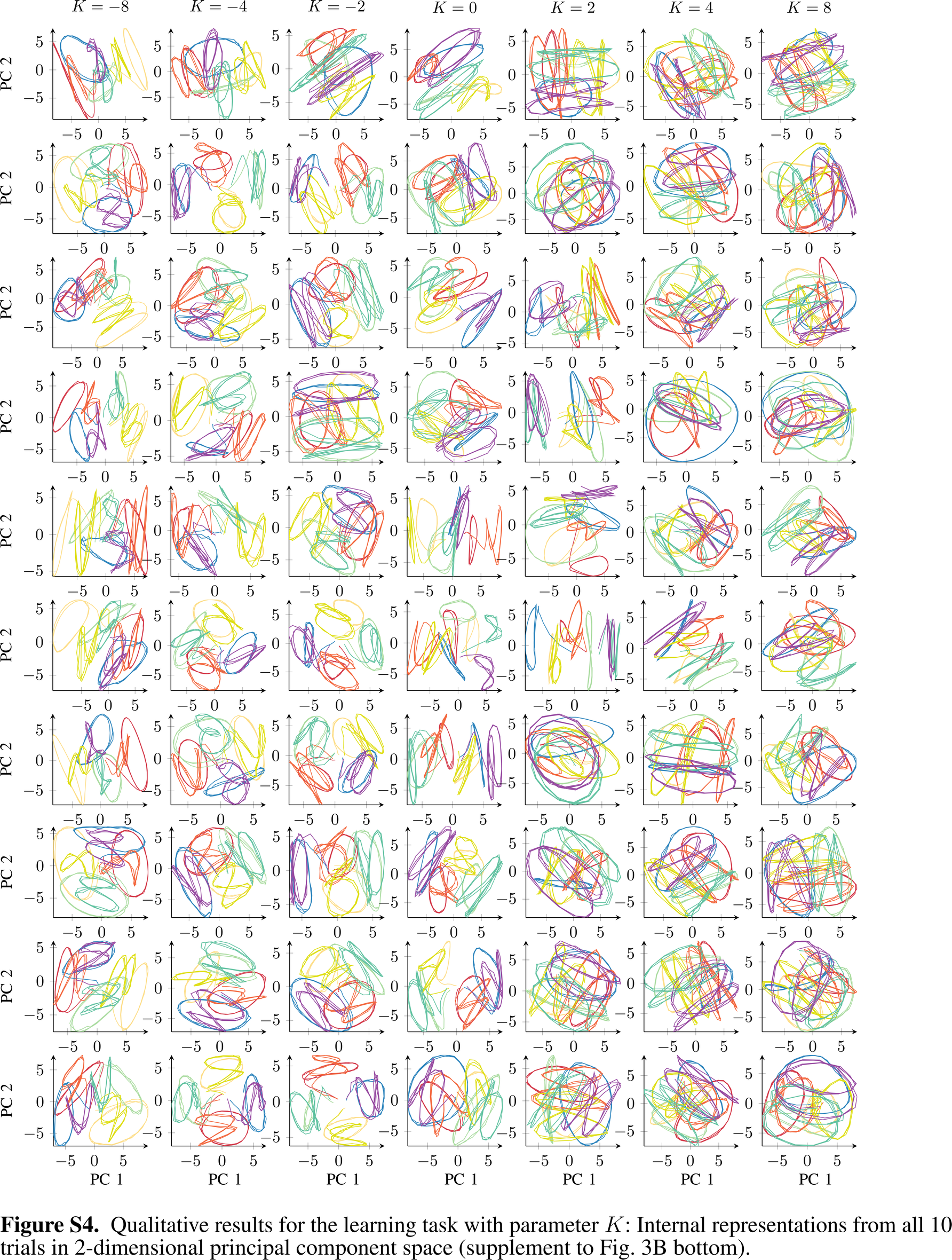

Supplement: Supplementary file 4 [file Image_4.TIF]

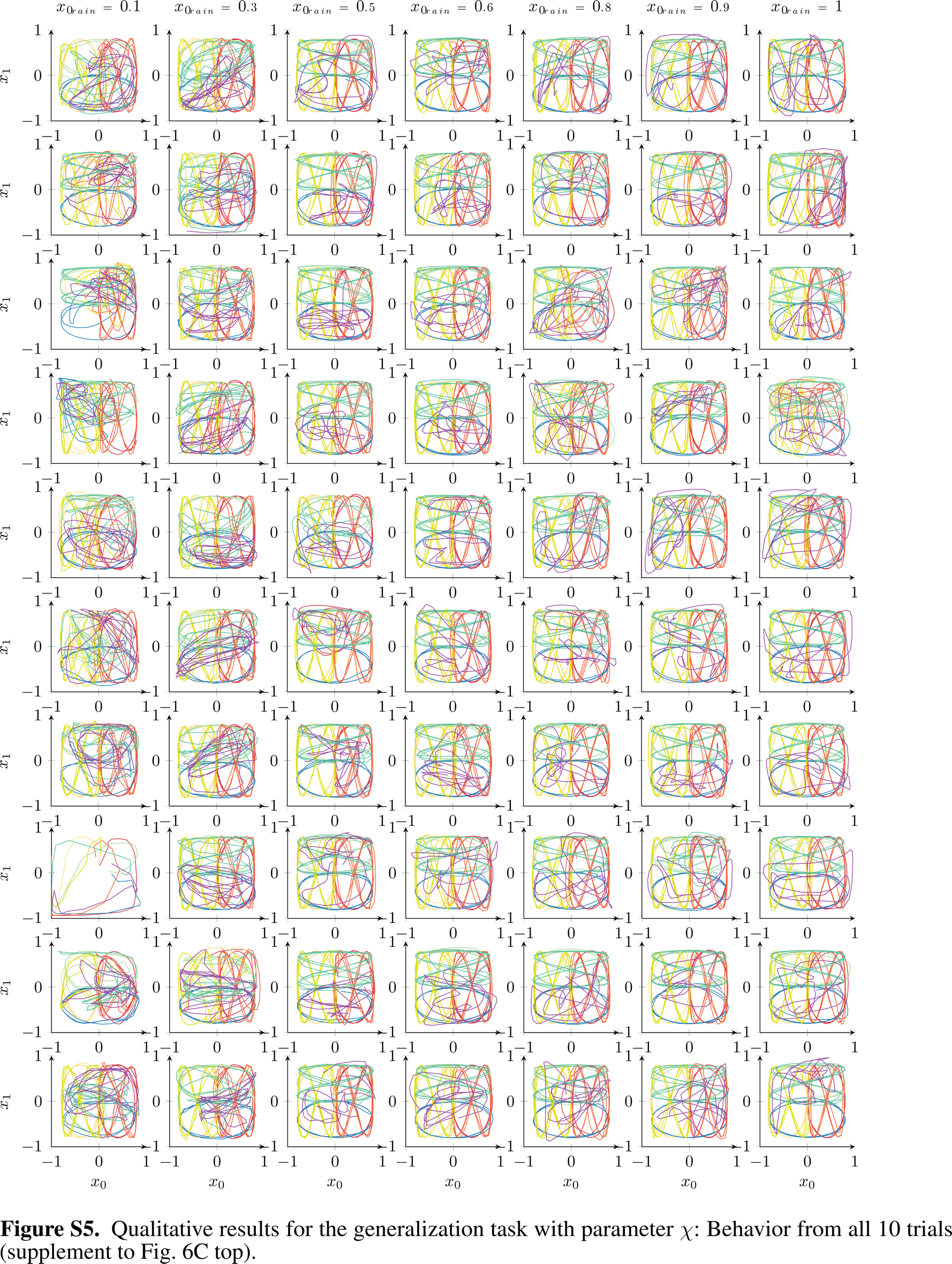

Supplement: Supplementary file 5 [file Image_5.TIF]

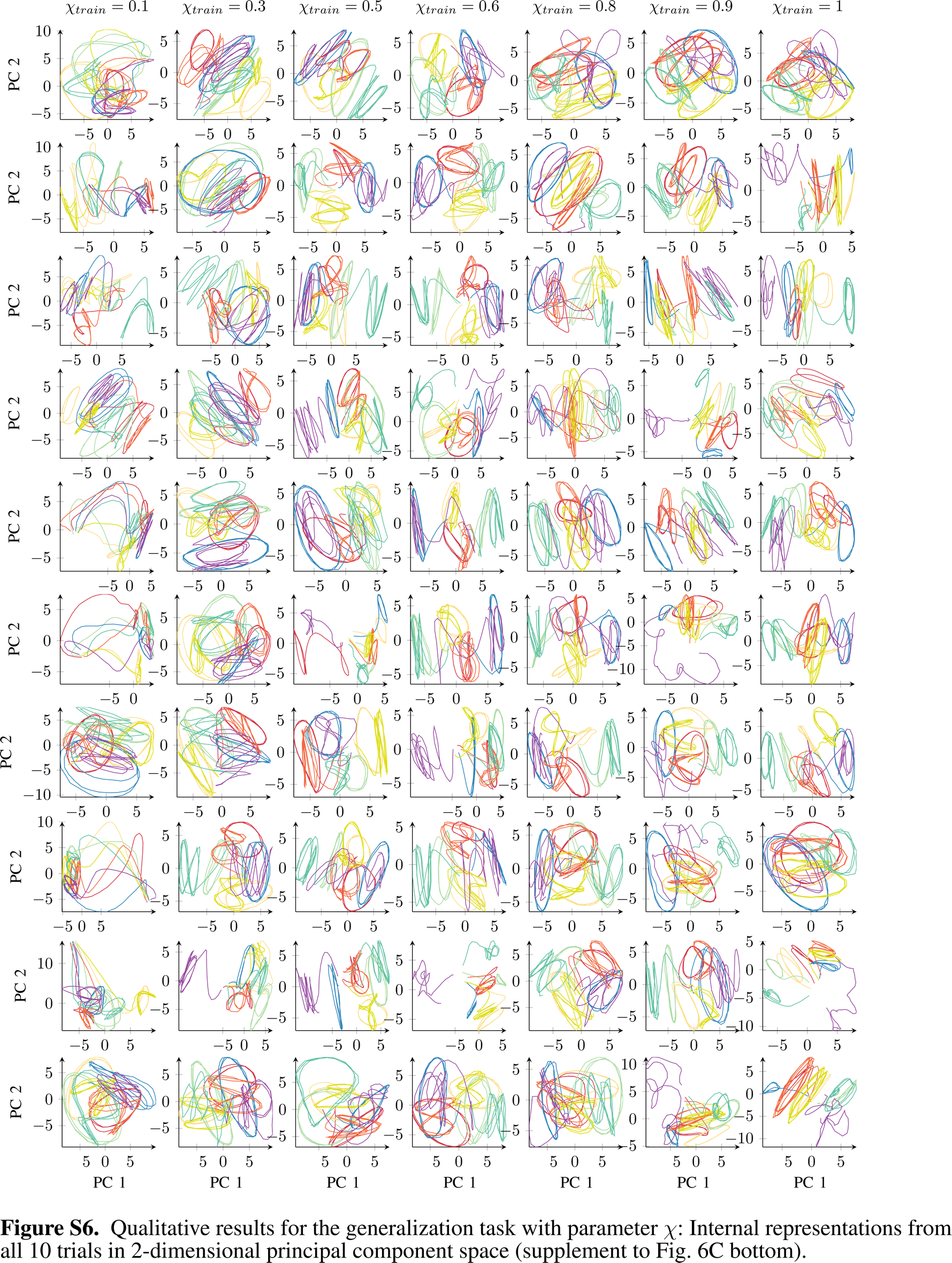

Supplement: Supplementary file 6 [file Image_6.TIF]

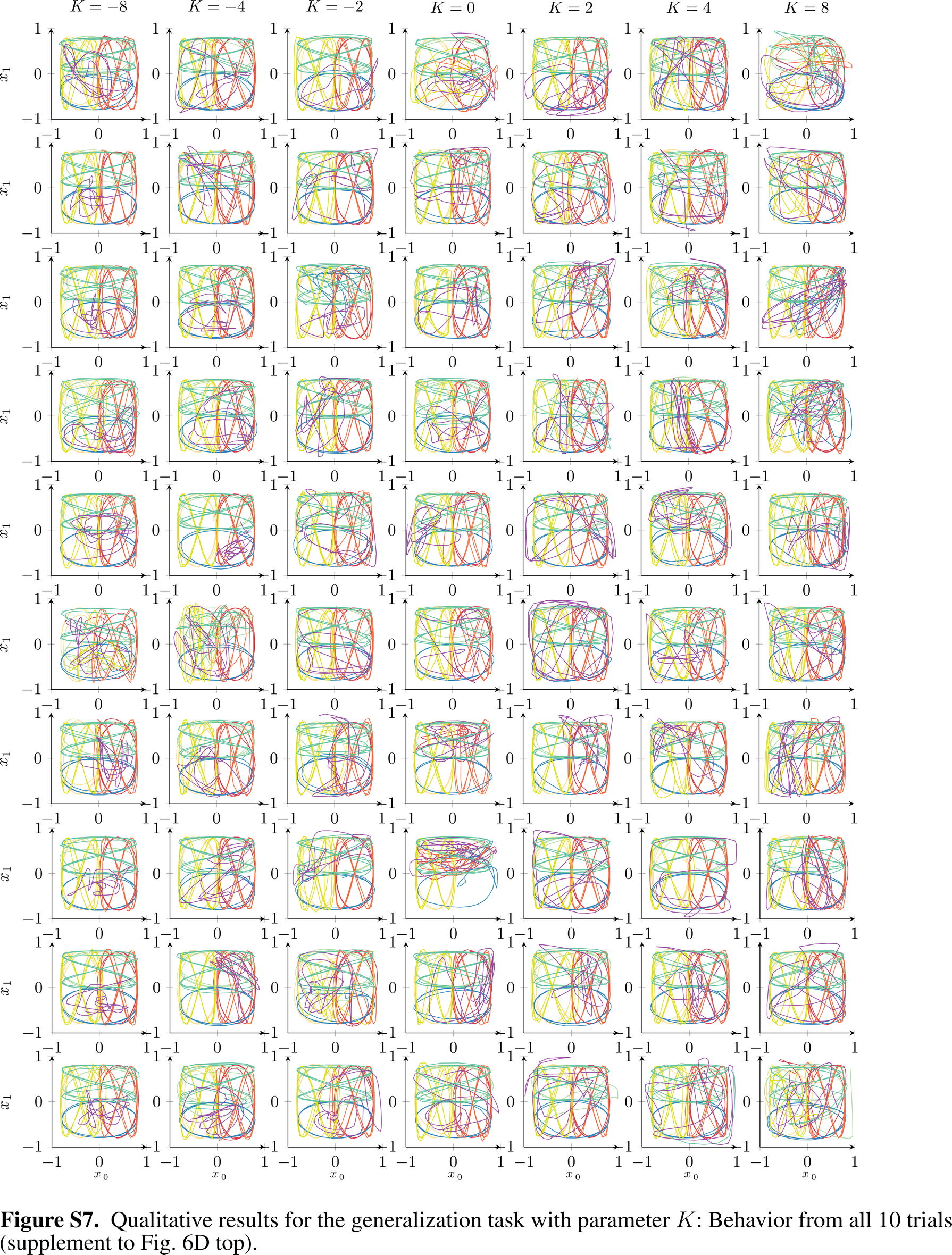

Supplement: Supplementary file 7 [file Image_7.TIF]

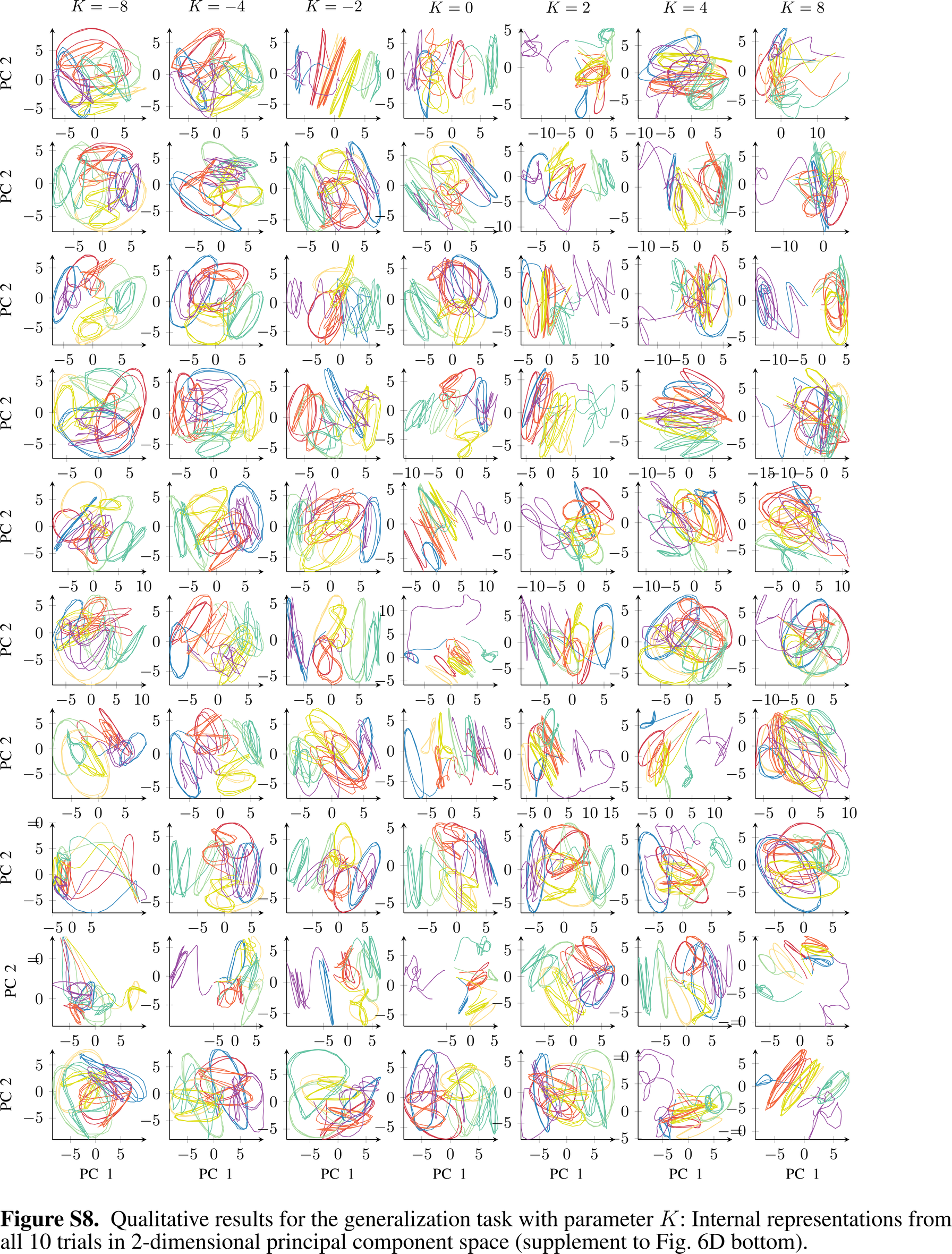

Supplement: Supplementary file 8 [file Image_8.TIF]
