# Supplementary material for: Deficits in Prediction Ability Trigger Asymmetries in Behavior and Internal Representation
Source: Front Psychiatry. 2020 Nov 20;11:564415. doi: 10.3389/fpsyt.2020.564415 (PMC7716881; doi:10.3389/fpsyt.2020.564415)
Supplement: Supplementary file 9 [file Table_1.pdf]

Table 1: Full results of significance tests (p-values) of the **inner–outer quotient differences** presented in Figure 4A. Statistical differences were evaluated on pairs of parameter conditions using the likelihood ratio test.

	**0.1**	**0.2**	**0.3**	**0.4**	**0.5**	**0.6**	**0.7**	**0.8**	**0.9**	**1.0**
**0.1**	–									
**0.2**		–		0.0534 .						
**0.3**			–			0.0223 *	0.0084 **	0.0065 **	0.0040 **	0.0246 *
**0.4**		0.0534 .		–		0.0025 **	0.0010 **	0.0006 ***	0.0005 ***	0.0064 **
**0.5**					–		0.0355 *	0.0265 *	0.0148 *	
**0.6**			0.0223 *	0.0025 **		–				
**0.7**			0.0084 **	0.0010 **	0.0355 *		–			
**0.8**			0.0065 **	0.0006 ***	0.0265 *			–		
**0.9**			0.0040 **	0.0005 ***	0.0148 *				–	
**1.0**			0.0246 *	0.0064 **						–
