# Supplementary material for: Deficits in Prediction Ability Trigger Asymmetries in Behavior and Internal Representation
Source: Front Psychiatry. 2020 Nov 20;11:564415. doi: 10.3389/fpsyt.2020.564415 (PMC7716881; doi:10.3389/fpsyt.2020.564415)
Supplement: Supplementary file 10 [file Table_2.pdf]

Table 2: Full results of significance tests (p-values) of the **performance on untrained data** presented in Figure 6A. Statistical differences were evaluated on pairs of parameter conditions using the likelihood ratio test.

	**0.1**	**0.2**	**0.3**	**0.4**	**0.5**	**0.6**	**0.7**	**0.8**	**0.9**	**1.0**
**0.1**	–		0.0063 **	0.0130 *			0.0680 .			
**0.2**		–	0.0534 .	0.0973 .						
**0.3**	0.0063 **	0.0534 .	–					0.0001 ***	0.0010 **	0.0010 **
**0.4**	0.0130 *	0.0973 .		–				0.0007 ***	0.0038 **	0.0028 **
**0.5**					–			0.0775 .		0.0633 .
**0.6**						–				
**0.7**	0.0680 .						–	0.0182 *	0.0463 *	0.0237 *
**0.8**			0.0001 ***	0.0007 ***	0.0775 .		0.0182 *	–		
**0.9**			0.0010 **	0.0038 **			0.0463 *		–	
**1.0**			0.0010 **	0.0028 **	0.0633 .		0.0237 *			–
