# Supplementary material for: Deficits in Prediction Ability Trigger Asymmetries in Behavior and Internal Representation
Source: Front Psychiatry. 2020 Nov 20;11:564415. doi: 10.3389/fpsyt.2020.564415 (PMC7716881; doi:10.3389/fpsyt.2020.564415)
Supplement: Supplementary file 11 [file Table_3.pdf]

Table 3: Full results of significance tests (p-values) of the **performance on trained data** presented in Figure 6A. Statistical differences were evaluated on pairs of parameter conditions using the likelihood ratio test.

	**0.1**	**0.2**	**0.3**	**0.4**	**0.5**	**0.6**	**0.7**	**0.8**	**0.9**	**1.0**
**0.1**	–			0.0411 *	0.0192 *	0.0137 *	0.0149 *	0.0198 *	0.0139 *	0.0202 *
**0.2**		–		0.0108 *	0.0009 ***	0.0002 ***	0.0003 ***	0.0011 **	0.0002 ***	0.0013 **
**0.3**			–	0.0916 .	0.0043 **	0.0006 ***	0.0008 ***	0.0054 **	0.0006 ***	0.0070 **
**0.4**	0.0411 *	0.0108 *	0.0916 .	–	0.0636 .	0.0029 **	0.0058 **	0.0829 .	0.0033 **	
**0.5**	0.0192 *	0.0009 ***	0.0043 **	0.0636 .	–					
**0.6**	0.0137 *	0.0002 ***	0.0006 ***	0.0029 **		–				
**0.7**	0.0149 *	0.0003 ***	0.0008 ***	0.0058 **			–			
**0.8**	0.0198 *	0.0011 **	0.0054 **	0.0829 .				–		
**0.9**	0.0139 *	0.0002 ***	0.0006 ***	0.0033 **					–	
**1.0**	0.0202 *	0.0013 **	0.0070 **							–
