# Supplementary material for: Deficits in Prediction Ability Trigger Asymmetries in Behavior and Internal Representation
Source: Front Psychiatry. 2020 Nov 20;11:564415. doi: 10.3389/fpsyt.2020.564415 (PMC7716881; doi:10.3389/fpsyt.2020.564415)
Supplement: Supplementary file 12 [file Table_4.pdf]

Table 4: Full results of significance tests (p-values) of the inner–outer quotient differences presented in Figure 4B. Statistical differences of the inner-outer quotient were evaluated on pairs of parameter conditions using the likelihood ratio test.

| | **-8** | **-4** | **-2** | **0** | **2** | **4** | **8** |
|---|---|---|---|---|---|---|---|
| **-8** | – | | | | 1.62e-07 *** | 4.55e-08 *** | 7.59e-07 *** |
| **-4** | | – | | | 2.50e-07 *** | 1.24e-07 *** | 1.29e-06 *** |
| **-2** | | | – | | 1.18e-05 *** | 1.25e-05 *** | 9.96e-05 *** |
| **0** | | | | – | 2.48e-05 *** | 2.88e-05 *** | 0.00023 *** |
| **2** | 1.62e-07 *** | 2.50e-07 *** | 1.18e-05 *** | 2.48e-05 *** | – | | 0.09657 . |
| **4** | 4.55e-08 *** | 1.24e-07 *** | 1.25e-05 *** | 2.88e-05 *** | | – | |
| **8** | 7.59e-07 *** | 1.29e-06 *** | 9.96e-05 *** | 0.00023 *** | 0.09657 . | | – |
