# Supplementary material for: Deficits in Prediction Ability Trigger Asymmetries in Behavior and Internal Representation
Source: Front Psychiatry. 2020 Nov 20;11:564415. doi: 10.3389/fpsyt.2020.564415 (PMC7716881; doi:10.3389/fpsyt.2020.564415)
Supplement: Supplementary file 13 [file Table_5.pdf]

Table 5: Full results of significance tests (p-values) of the **performance on untrained data** presented in Figure 6A. Statistical differences were evaluated on pairs of parameter conditions using the likelihood ratio test.

	**-8**	**-4**	**-2**	**0**	**2**	**4**	**8**
**-8**	–		3.64e-05 ***	1.43e-05 ***	3.60e-05 ***	1.50e-06 ***	4.50e-06 ***
**-4**		–	0.0217 *	0.0026 **	0.0018 **	0.0007 ***	0.0045 **
**-2**	3.64e-05 ***	0.0217 *	–		0.0655 .	0.0490 *	
**0**	1.43e-05 ***	0.0026 **		–			
**2**	3.60e-05 ***	0.0018 **	0.0655 .		–		
**4**	1.50e-06 ***	0.0007 ***	0.0490 *			–	
**8**	4.50e-06 ***	0.0045 **					–
