# Supplementary material for: Deficits in Prediction Ability Trigger Asymmetries in Behavior and Internal Representation
Source: Front Psychiatry. 2020 Nov 20;11:564415. doi: 10.3389/fpsyt.2020.564415 (PMC7716881; doi:10.3389/fpsyt.2020.564415)
Supplement: Supplementary file 14 [file Table_6.pdf]

Table 6: Full results of significance tests (p-values) of the **performance on trained data** presented in Figure 6A. Statistical differences were evaluated on pairs of parameter conditions using the likelihood ratio test.

	**-8**	**-4**	**-2**	**0**	**2**	**4**	**8**
**-8**	–						
**-4**		–					0.0563 .
**-2**			–				
**0**				–			
**2**					–		0.0589 .
**4**						–	0.0498 *
**8**		0.0563 .			0.0589 .	0.0498 *	–
